# Supplementary figures and images for: Genome-scale CRISPR screen identifies TMEM41B as a multi-function host factor required for coronavirus replication
Source: PLoS Pathog. 2021 Dec 6;17(12):e1010113. doi: 10.1371/journal.ppat.1010113 (PMC8675922; doi:10.1371/journal.ppat.1010113)

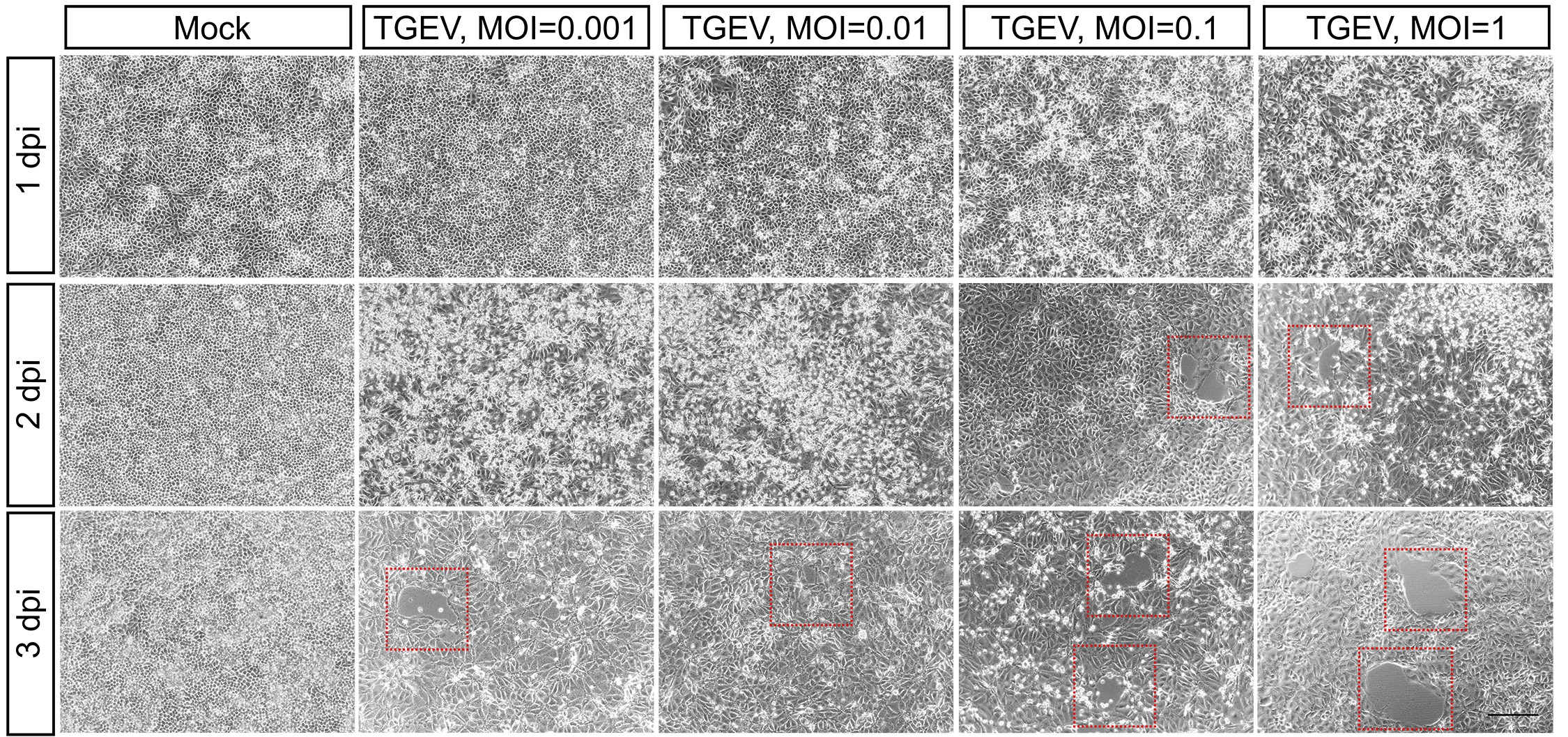

Supplement: S1 Fig — The red boxes indicate TGEV-induced cytopathic effects (CPE) in PK-15 cells infected with TGEV at MOIs of 0.001, 0.01, 0.1, or 1. The mock was non-infected TGEV cells used as a negative control. Scale bar, 200 μm. MOI, multiplicity of infection; dpi, days post-infection; TGEV, Transmissible gastroenteritis virus. (TIF) [file ppat.1010113.s001.tif]

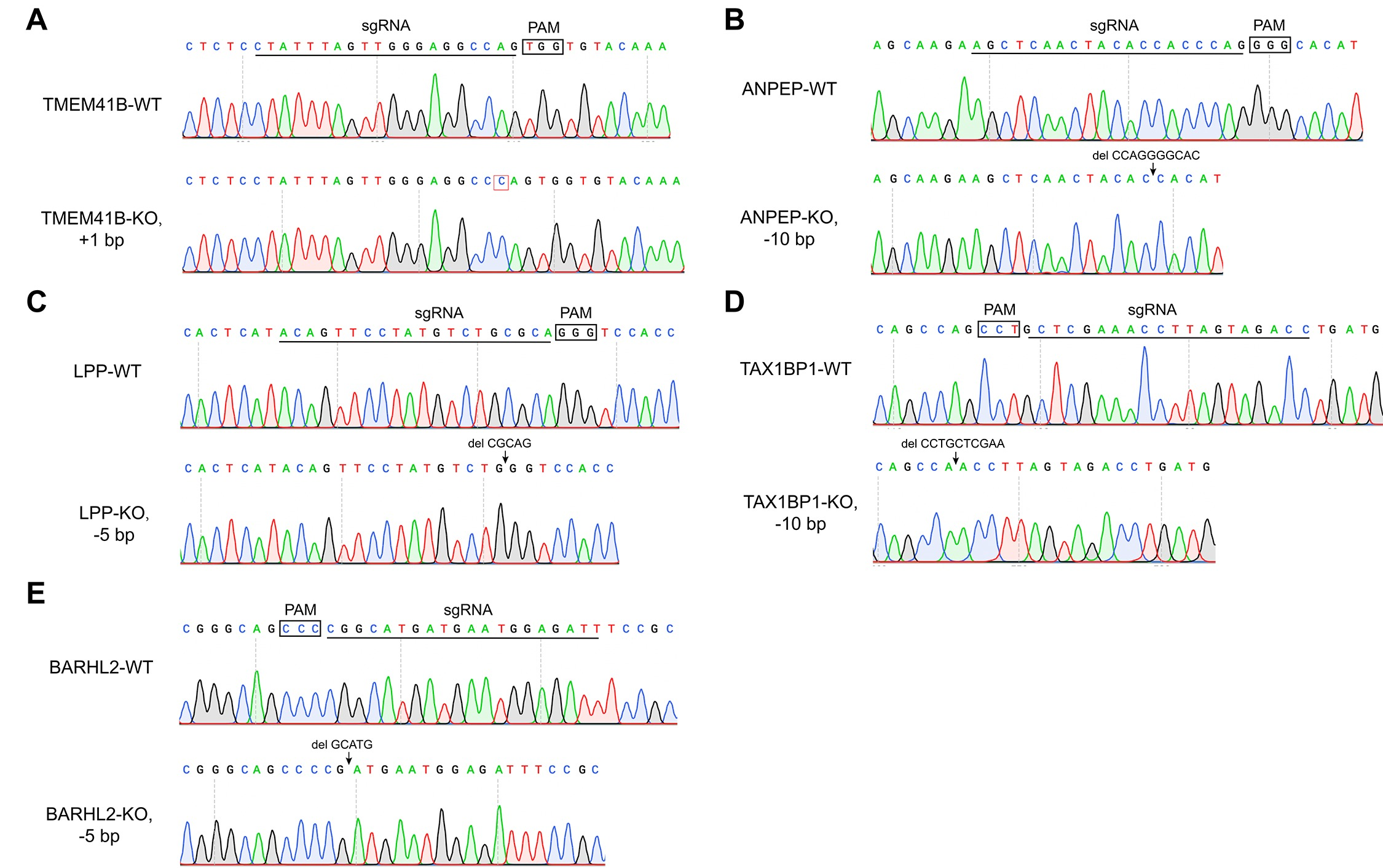

Supplement: S2 Fig — DNA sequence analysis showing the presence of the mutation in clonal KO cells of TMEM41B (A), ANPEP (B), LPP (C), TAX1BP1 (D), and BARHL2 (E). The underline indicates the deleted bases in the KO cells. The black box indicates the PAM sites. PAM: protospacer adjacent motif; sgRNA, small guide RNA; WT, wild-type; KO, knockout; del, deletion; bp, base pairs. (TIF) [file ppat.1010113.s002.tif]

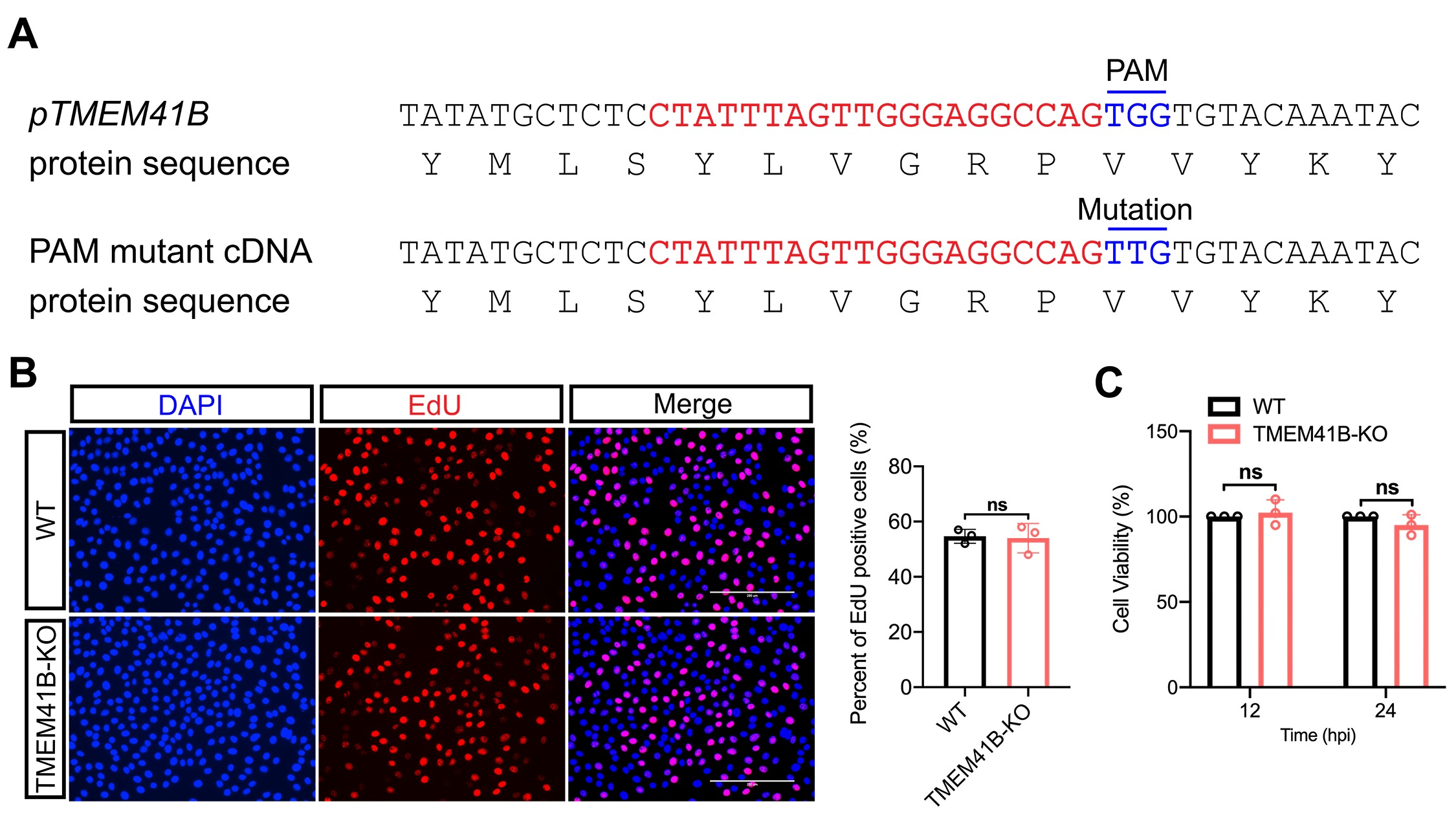

Supplement: S3 Fig — (A) Design of CRISPR-resistant pTMEM41B sequences. To abolish cleavage by sgRNA and Cas9 in TMEM41B KO cells, a specific point mutation in PAM sequence, which does not alter the amino acid was introduced into the TMEM41B coding sequences. (B) There was no significant change in the proportion of EdU positive cells in clonal TMEM41B KO cell lines compared to WT cells. Left: representative pictures. Right: quantification of EdU positive cells. Quantitative analysis of immunofluorescence was used by Image J software. (C) TMEM41B KO and PK-15 WT cells were seeded into 96-well plates to evaluate cell proliferation by MTS assays at 12 hpi and 24 hpi. Scale bar, 200 μm. Data are represented as means ± S.D.; n = 3, ns: no significant. pTMEM41B, porcine TMEM41B gene; WT, wild-type; KO, knockout; PAM, protospacer adjacent motif; hpi, hours post-infection; DAPI, 4’,6-diamidino-2-phenylindole. ns, no significant. P values were determined by two-sided Student’s t-test. (TIF) [file ppat.1010113.s003.tif]

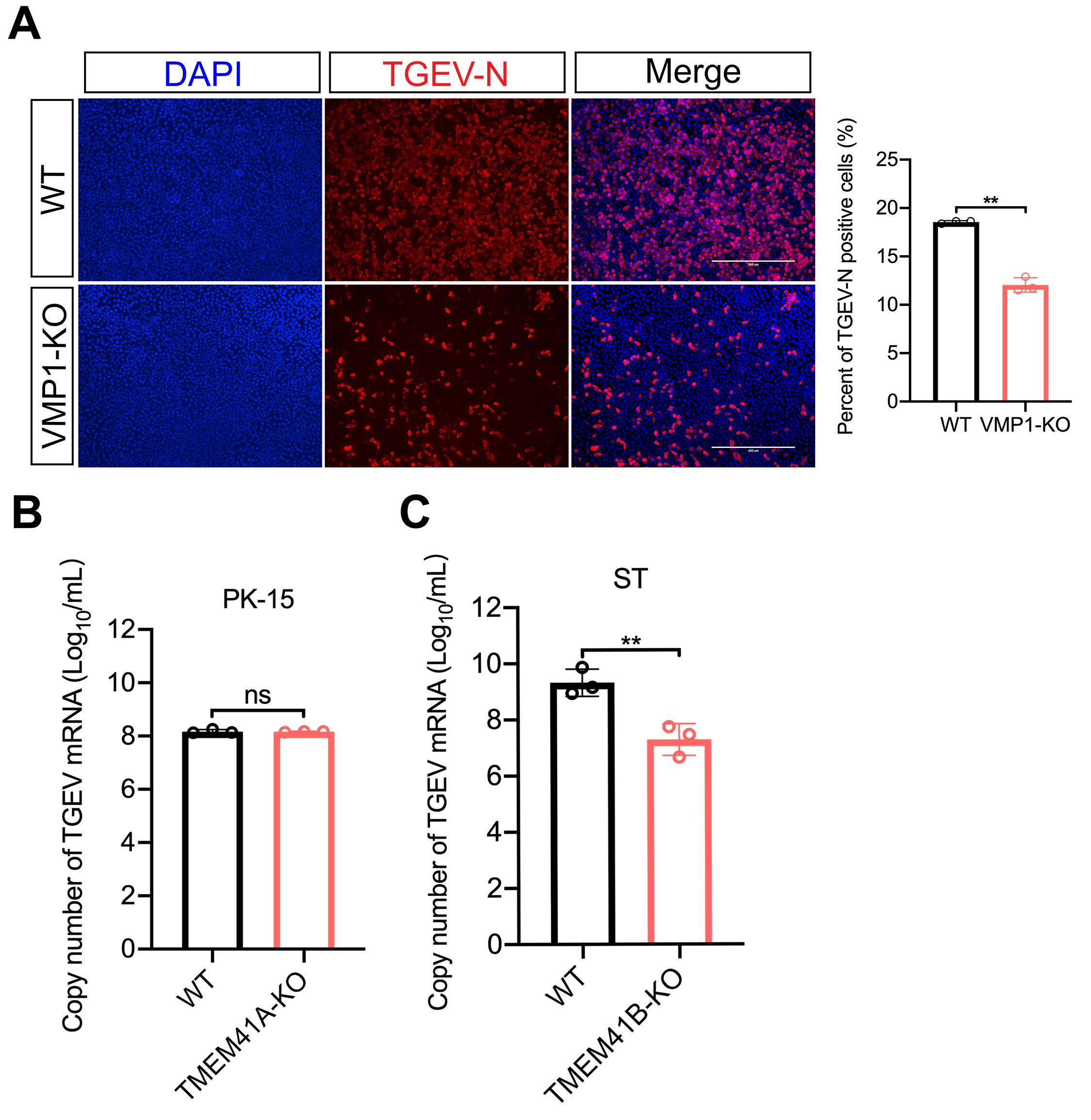

Supplement: S4 Fig — (A) WT cells and VMP1 KO PK-15 cells were infected with TGEV (MOI = 1) for 24h and used for immunofluorescence assays. Left: representative pictures. Right: quantification of TGEV N positive cells. Quantitative analysis of immunofluorescence was conducted with Image J software. Scale bar, 400 μm. (B) WT cells and TMEM41A KO PK-15 cells were infected with TGEV (MOI = 1) for 24h and used for qRT-PCR to measure the TGEV N copy number. (C) WT cells and TMEM41B KO ST cells were infected with TGEV (MOI = 1) for 24 h and used for qRT-PCR to measure TGEV N copy number. WT, wild-type; KO, knockout; DAPI, 4’,6-diamidino-2-phenylindole; ST, porcine Sertoli cells. **P < 0.001; ns, no significant. P values were determined by two-sided Student’s t-test. (TIF) [file ppat.1010113.s004.tif]

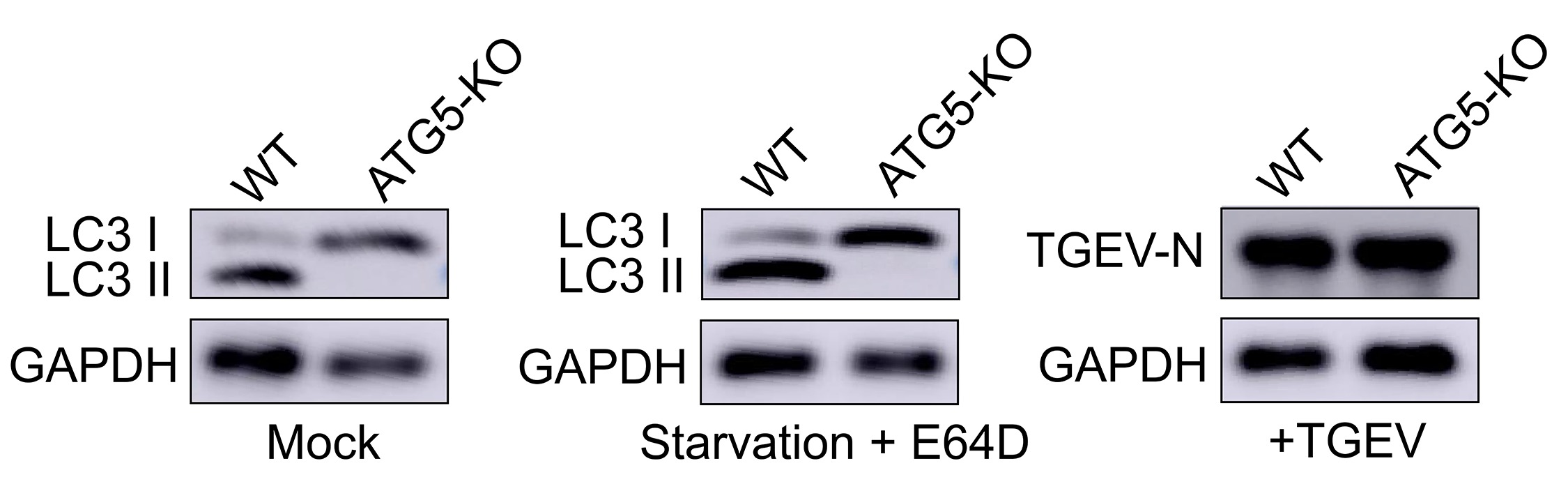

Supplement: S5 Fig — (Left) Western blot analysis of LC3-I and LC3-II in untreated WT and ATG5 KO cells; (Middle) Western blot analysis of LC3-I and LC3-II in WT and ATG5 KO cells after EBSS medium (EBSS) starvation and E64d supplementation for 6 h; (Right) Western blot analysis of TGEV N in WT and ATG5 KO cells following infection with TGEV (MOI = 1) at 12 hpi. GAPDH was shown as a loading control. WT, wild-type; KO, knockout; GAPDH, glyceraldehyde-3-phosphate dehydrogenase; TGEV, Transmissible gastroenteritis virus infected cells; Mock, uninfected cells; E64D, aloxistatin; ATG5, autophagy related gene 5. (TIF) [file ppat.1010113.s005.tif]

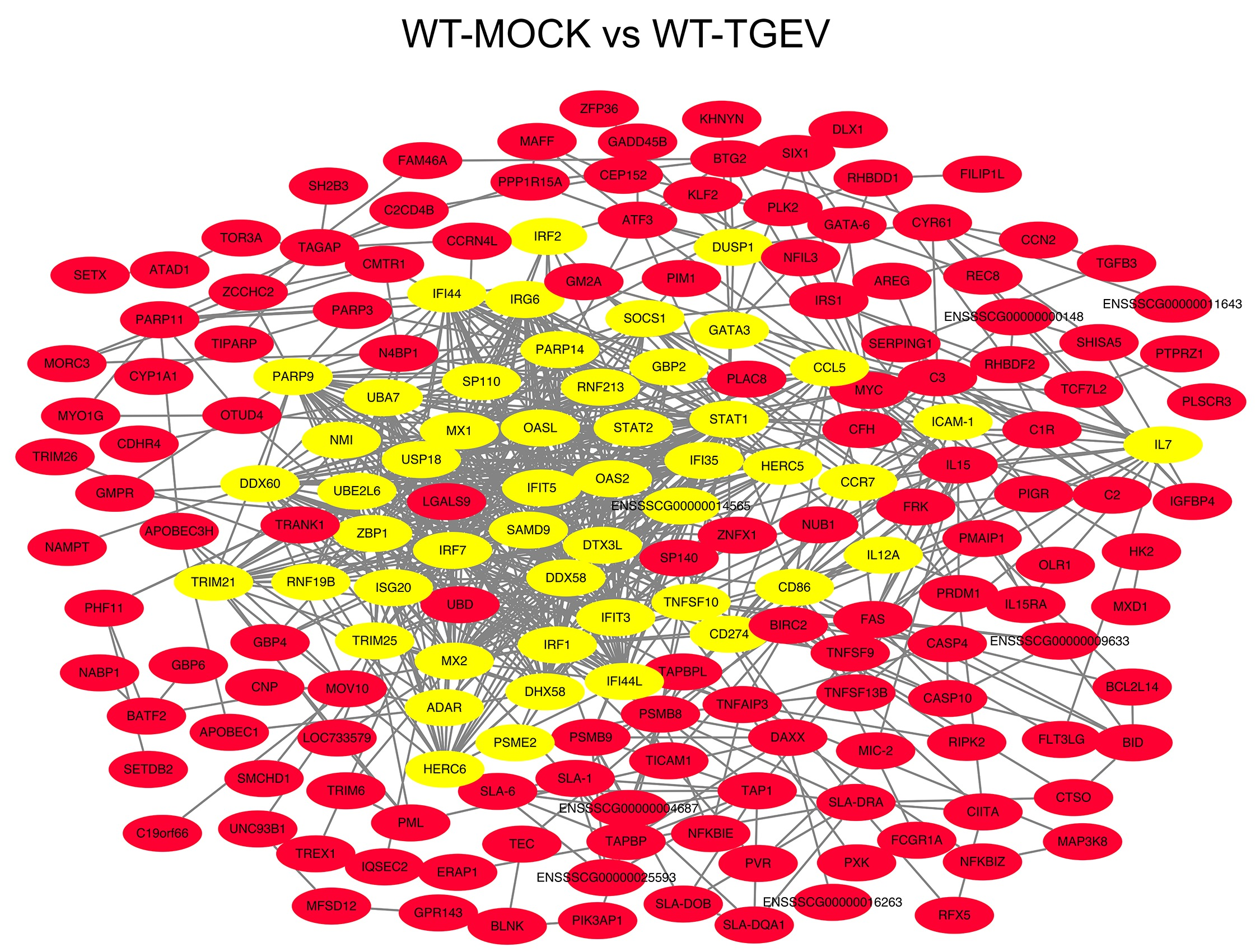

Supplement: S6 Fig — Differentially expressed genes in the "interferon signaling" pathway were marked with yellow. WT, wild-type; TGEV, Transmissible gastroenteritis virus infected cells; Mock, uninfected cells. (TIF) [file ppat.1010113.s006.tif]

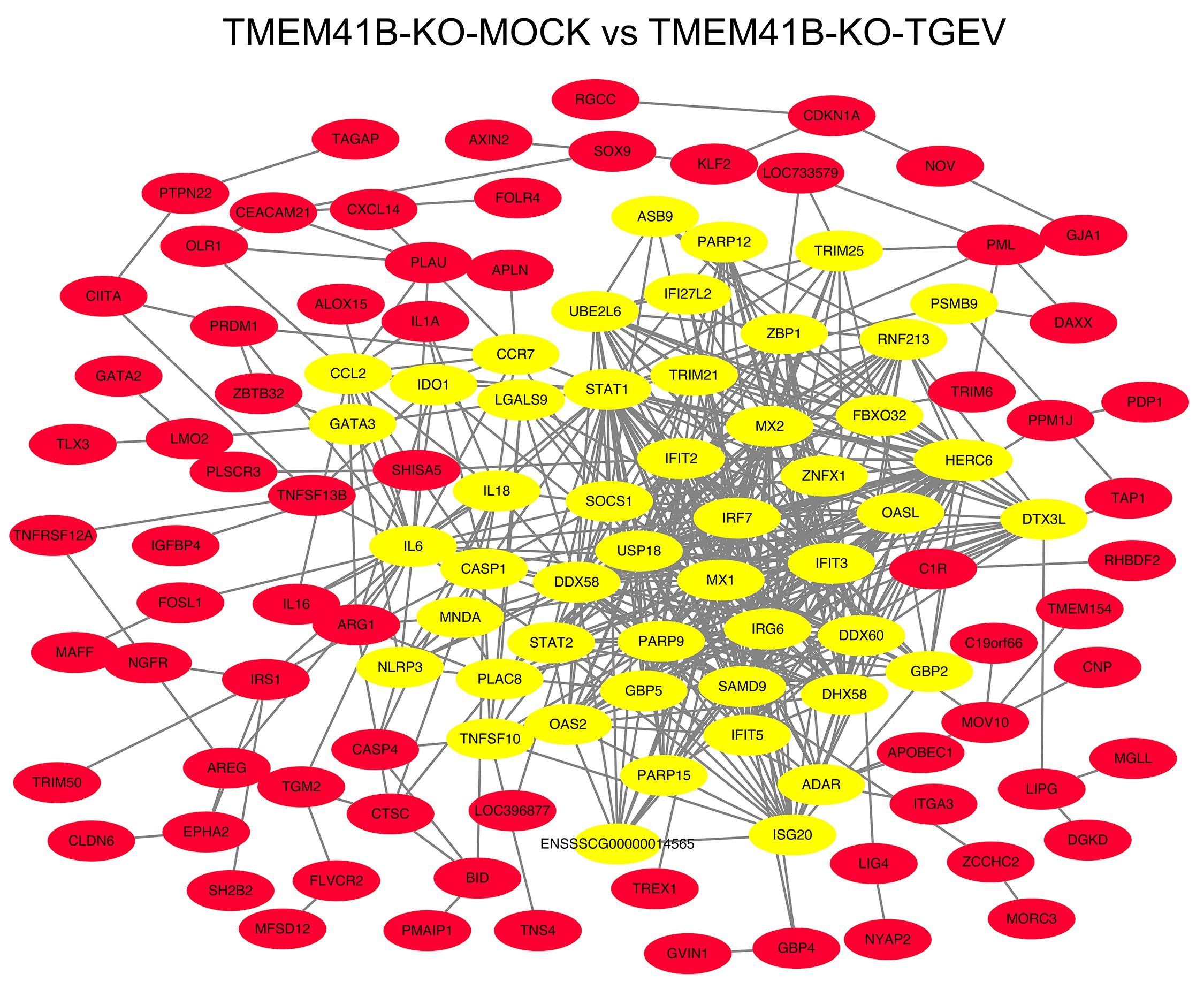

Supplement: S7 Fig — Differentially expressed genes in the "interferon signaling" pathway were marked with yellow. KO, knockout; TGEV, Transmissible gastroenteritis virus infected cells; Mock, uninfected cells. (TIF) [file ppat.1010113.s007.tif]

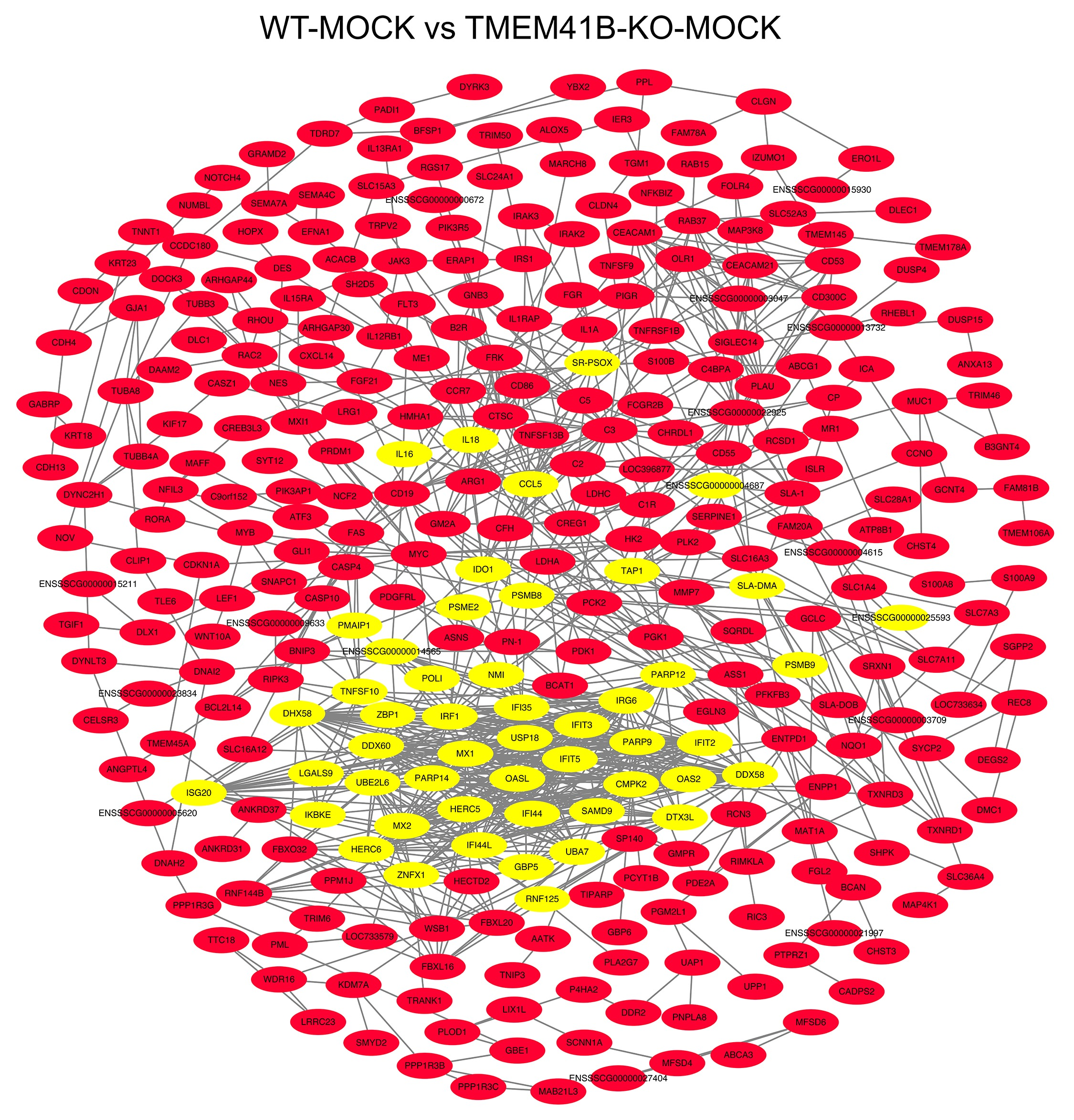

Supplement: S8 Fig — Differentially expressed genes in the "interferon signaling" pathway were marked with yellow. WT, wild-type; KO, knockout; Mock, uninfected cells. (TIF) [file ppat.1010113.s008.tif]

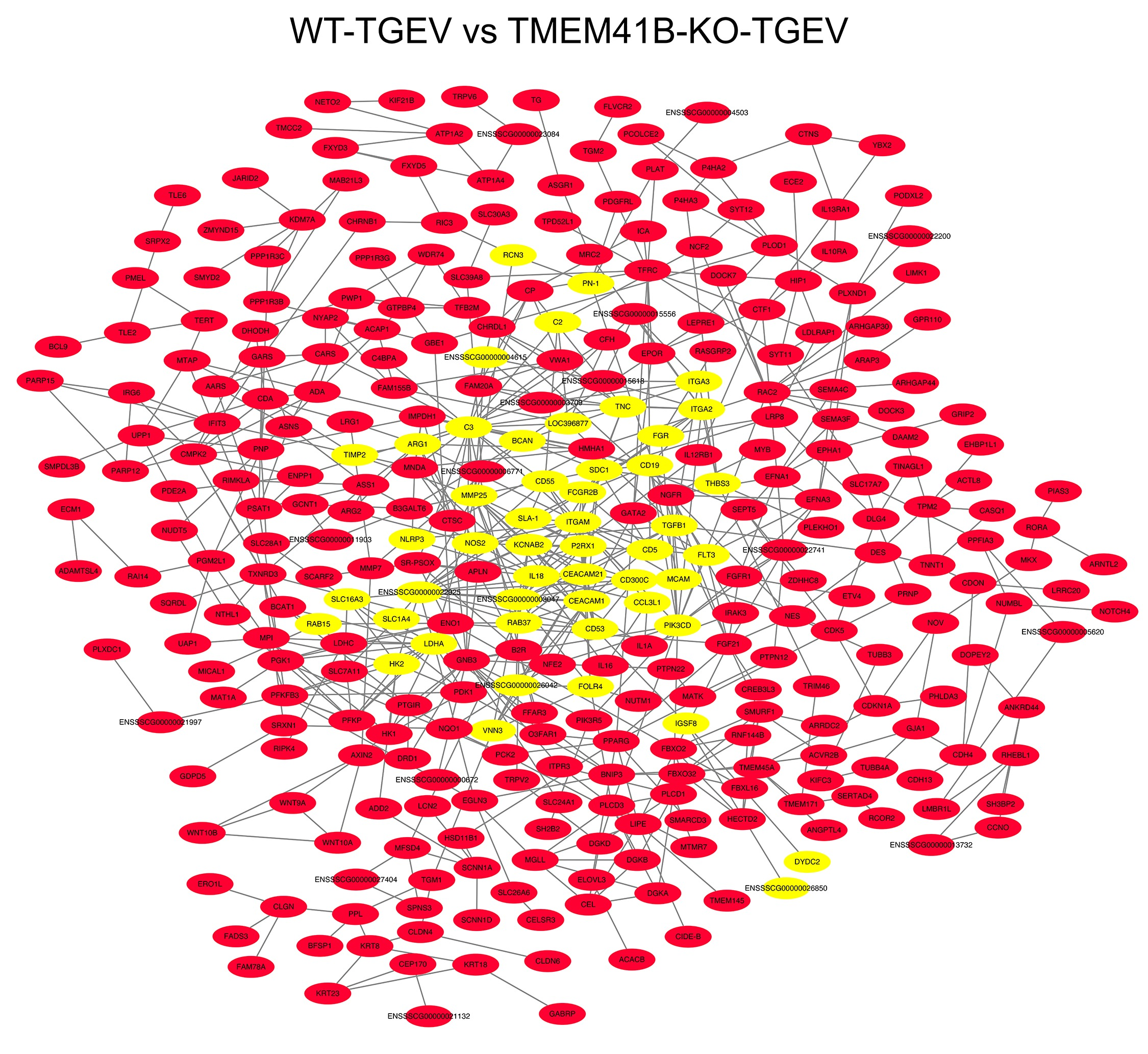

Supplement: S9 Fig — Differentially expressed genes in the "interferon signaling" pathway were marked with yellow. WT, wild-type; KO, knockout; TGEV, Transmissible gastroenteritis virus infected cells. (TIF) [file ppat.1010113.s009.tif]

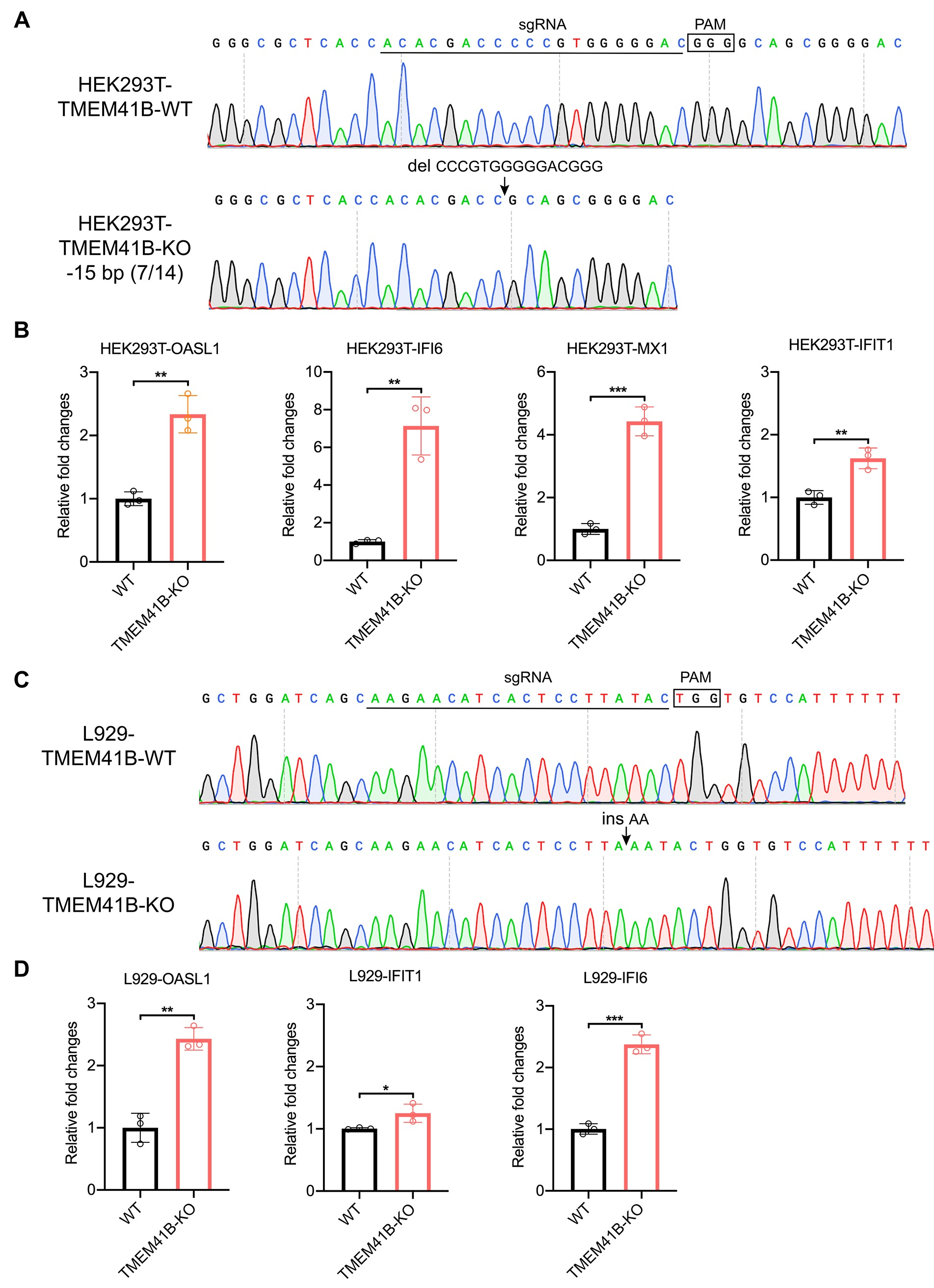

Supplement: S10 Fig — (A and C) DNA sequence analysis showed that the presence of the mutation in (A) HEK293T and (C) L929 single clone originated TMEM41B KO cells. (B and D) Detection of mRNA expression of the ISGs in (B) TMEM41B-KO HEK293T and (D) TMEM41B-KO L929 cells by RT-qPCR. sgRNA, small guide RNA; PAM, protospacer adjacent motif; bp, base pairs; ins, insertion; del, deletion; WT, wild-type; KO, knockout. *P < 0.05; **P < 0.01; ***P < 0.001. P values were determined by two-sided Student’s t-test. Data are representative of at least three independent experiments. (TIF) [file ppat.1010113.s010.tif]

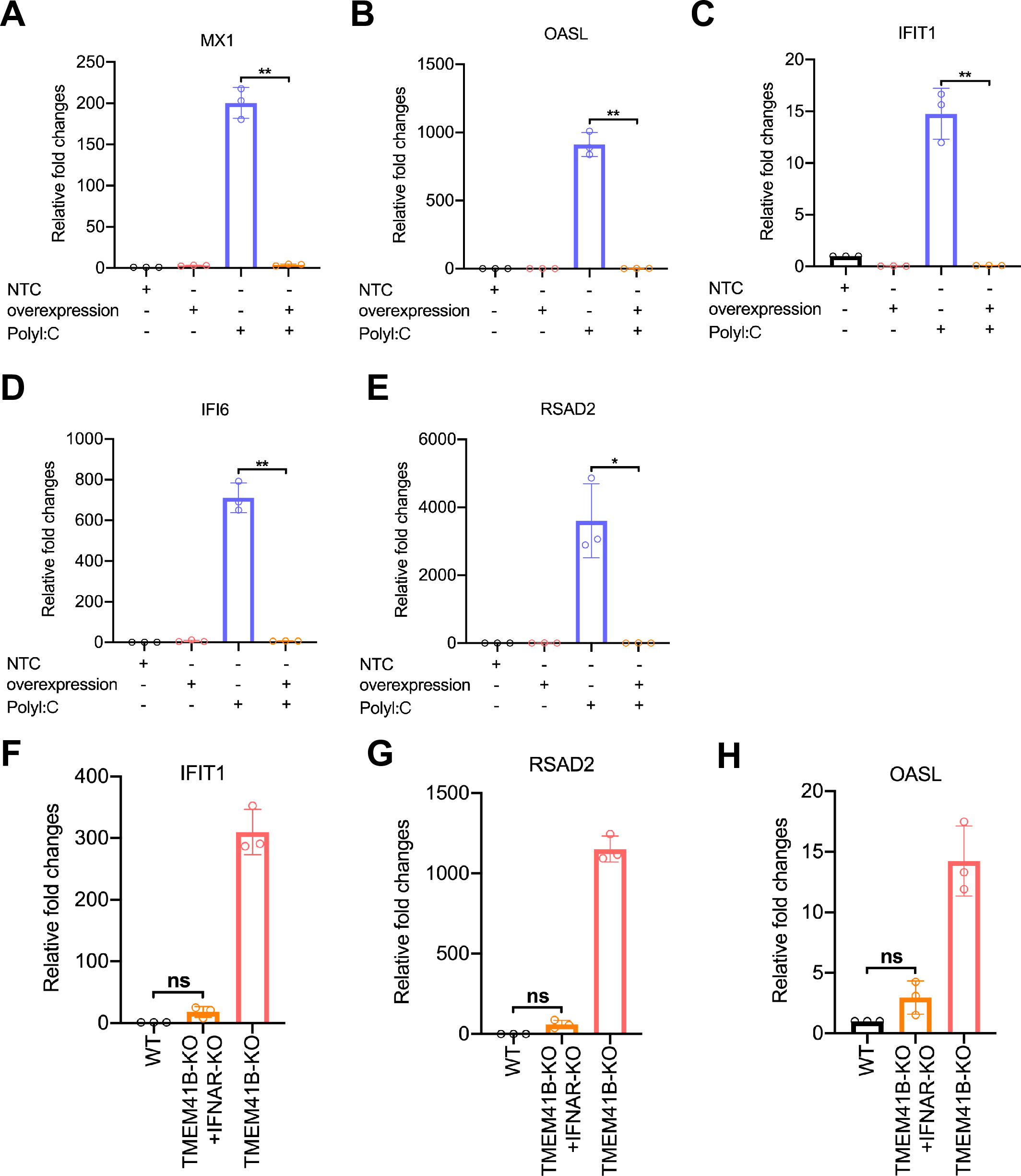

Supplement: S11 Fig — (A-E) mRNA levels of the ISGs (A) MX1, (B) OASL, (C) IFIT1, (D) IFI6 and (E) RASD2 on PK-15 cells or PK-15 cells overexpressing TMEM41B with or without PolyI:C stimulation. (F-H) mRNA levels of ISGs (F) IFIT1, (G) RASD2 and (H) OASL in TMEM41B and IFNAR double KO cells were reduced to WT levels compared with TMEM41B KO cells. Data are representative of at least three independent experiments. NTC, negative control; PolyI:C, Polyinosinic: polycytidylic acid; WT, wild-type; KO, knockout. *P < 0.05; **P < 0.01; ***P < 0.001; ns, no significant. P values were determined by two-sided Student’s t-test. Data are representative of at least three independent experiments. (TIF) [file ppat.1010113.s011.tif]

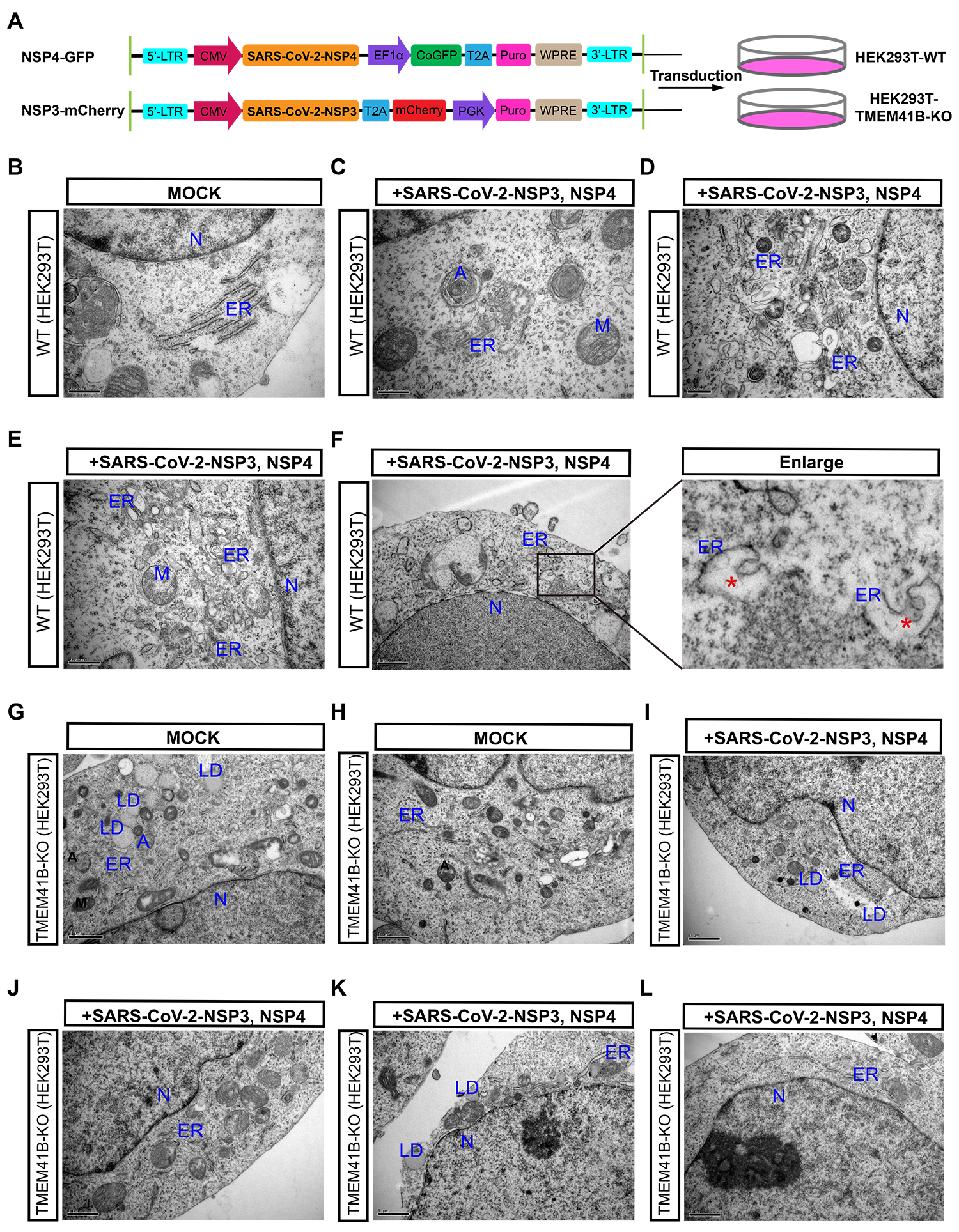

Supplement: S12 Fig — (A) Schematic illustration of the backbone of SARS-CoV-2 Nsps plasmids. First, we generated TMEM41B KO HEK293T cell lines. Subsequently, we constructed PLV-mCherry-SARS-CoV-2-NSP3 and PLV-eGFP-SARS-CoV-2-NSP4 lentiviral expression vectors. After co-infection of PLV-mCherry-SARS-CoV-2-NSP3 and PLV-eGFP-SARS-CoV-2-NSP4 lentivirals in TMEM41B KO and WT HEK293T cells, the double immunofluorescence cells (mCherry and eGFP) were sorted by flow cytometer. (B-F) (B) HEK293T MOCK-treated cells and (C-F) SARS-CoV-2 NSP3 and NSP4 co-expressed cells were fixed for TEM analysis. (C) DMVs and (D-F) large deformation of ER can be observed. Red arrows indicate curved ERs. (G-H) HEK293T-TMEM41B-KO MOCK treatment cells (G and H) and SARS-CoV-2 NSP3 and NSP4 co-expressed cells (I-L) were fixed for TEM analysis. Mock, untreated control cells; +SARS2-CoV2-NSP3, NSP4, co-overexpression of NSP3 and NSP4 from SARS-CoV-2; WT, wild-type; KO, knockout; M, Mitochondria; ER, Endoplasmic reticulum; N, Nucleus; LD, lipid droplet; A, autophagosome; LTR, long terminal repeat; CMV, cytomegalovirus promoter; WPRE, Woodchuck Hepatitis Virus (WHP) Posttranscriptional Regulatory Element; GFP; green fluorescent protein; mCherry, mCherry fluorescent protein. Mock, uninfected cells. (TIF) [file ppat.1010113.s012.tif]

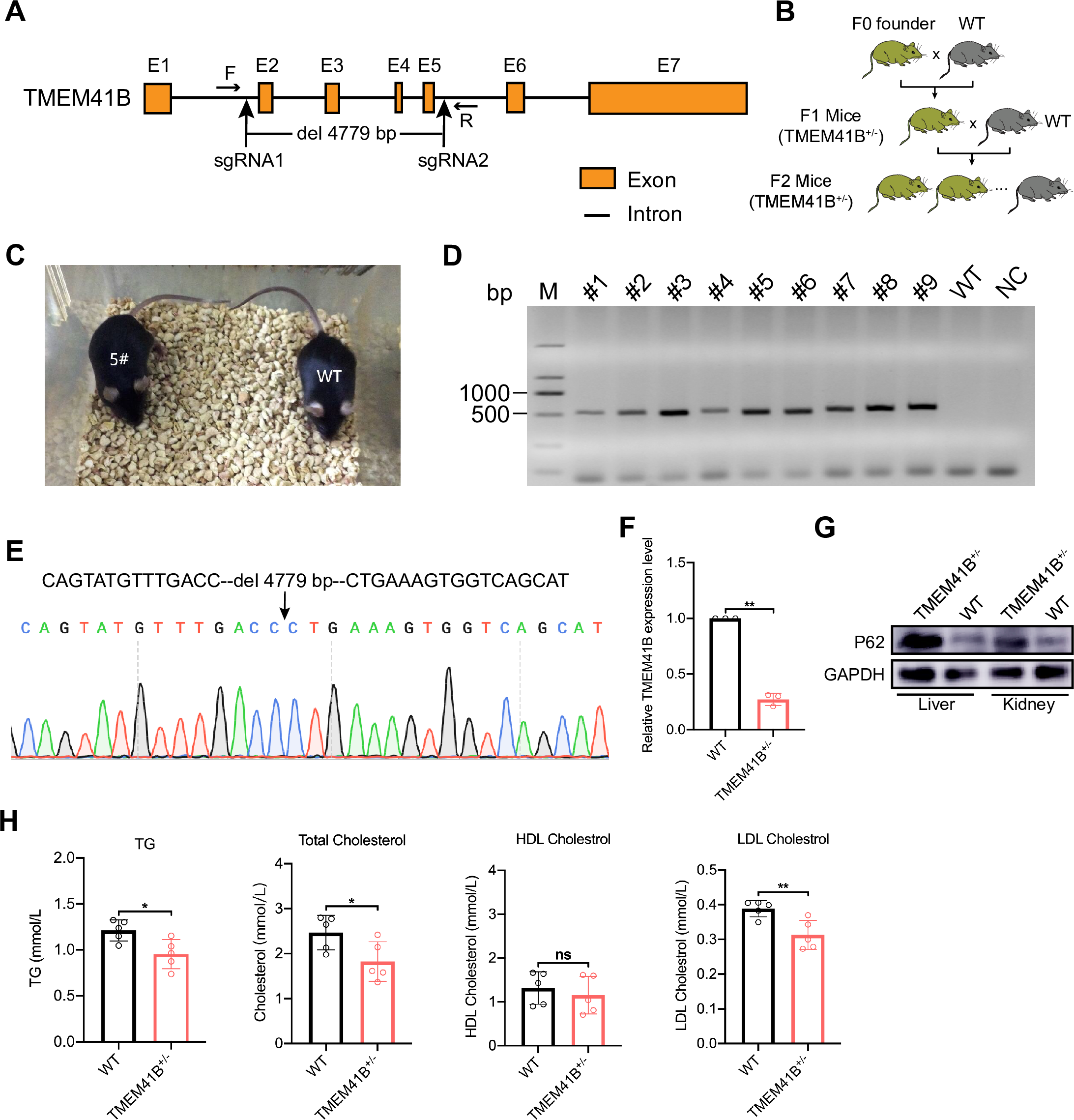

Supplement: S13 Fig — (A) A schematic diagram of generation of F2 TMEM41B-deficient mice. (B and C) There was no significant morphological difference between F2 generation TMEM41B+/− mice and WT (C57BL/6N) mice. (D) Genotyping of F2 generation TMEM41B+/− mice using genomic DNA from tail clips. (E) Sanger sequencing of PCR products from F2 generation TMEM41B+/−mice. (F) The relative TMEM41B gene expression level of F2 generation TMEM41B+/− mice liver tissue was reduced by about 70% compared with WT mice liver tissue. (G) Western blot assays analysis the autophagy maker p62 protein of liver and kidney tissue from WT and TMEM41B+/− mice with starvation treatment. (H) The serum lipid concentrations of WT and TMEM41B+/− mice (before MHV infection) were measured by corresponding reagent kit: TG (Rayto, S03027); CHO (Rayto, S03042); HDL (Rayto, S03025); LDL (Rayto, S03029). F, forward primer; R, reverse primer; WT, wild-type; NC, negative control; del, deletion, E1-7, exons from 1 to 7; bp, base pairs; M, DNA marker; sgRNA, small guide RNA; F0, founder generation mice; F1, F1 generation mice; F2, F2 generation mice; TG, triglyceride; CHO, cholesterol; HDL, High-density lipoprotein; LDL, Low-density lipoprotein. *P < 0.05; **P < 0.01; ***P < 0.001; ns, no significant. P values were determined by two-sided Student’s t-test. Data are representative of at least three independent experiments. (TIF) [file ppat.1010113.s013.tif]
