# Supplementary material for: Genome-scale CRISPR screen identifies TMEM41B as a multi-function host factor required for coronavirus replication
Source: PLoS Pathog. 2021 Dec 6;17(12):e1010113. doi: 10.1371/journal.ppat.1010113 (PMC8675922; doi:10.1371/journal.ppat.1010113)
Supplement: Attachment — Submitted filename: Response to reviewers.docx [file ppat.1010113.s018.docx]

**Response to reviewers**

Reviewer #1 (Remarks to the Author):

COMMENT:

Multiple independent studies using genome-wide CRISPR-Cas9 screens have identified TMEM41B as playing critical roles in autophagy and lipid mobilization. Subsequent screens to identify host factors required for viral replication identified TMEM41B as important for the replication of diverse coronaviruses and flaviviruses. The authors extend the requirement of TMEM41B to a member of the Alphacoronavius family, TGEV, in a different host organism, Sus scrofa (Pig). Their findings are mostly in line with those previously reported in Hoffmann et al 2021. Novel findings from this study include the *in vivo* work and a potentially interesting association between TMEM41B and innate immune regulation. This manuscript is the first to demonstrate the functional relevance of TMEM41B and coronavirus disease pathogenesis *in vivo*. The authors propose a novel function for TMEM41B in regulating the innate immune response, however, additional evidence is needed to substantiate this function of TMEM41B.

RESPONSE:

We thank the reviewers for pointing out this issue. In the following sections, you will find our responses to each of your points and suggestions.

Major points (1): If TMEM41B is the primary cause of the TGEV replication defect observed in the TMEM41B KO cells, why is there still such a drastic difference in TGEV replication when TMEM41B expression is restored in KO cells? The authors should include an explanation as to why TGEV replication is not rescued in the TMEM41B reconstituted cells. See Figure 2g-j: The relative levels of TMEM41B expression between TMEM41B-KO-rescue (figure 2g) and PK-15-overexpression (figure 2j) look comparable (relative to WT-NTC and PK-15-NTC, respectively) yet the TGEV mRNA detected in the RT-qPCR looks to differ by around 4 logs (figure 2h and j). TMEM41B-KO-rescue cells had 3 logs less TGEV mRNA detected relative to the WT-NTC (figure 2h).

RESPONSE:

Thank you bringing this issue to our attention. In our previous rescue experiment, we only transiently transfected pcDNA3.1-TMEM41B plasmid into TMEM41B KO cells. To check why TGEV replication is partially rescued in the TMEM41B reconstituted cells, an immunofluorescence assay was performed. Due to the low transfection efficiency, we found that only partially overexpressed cells can support TGEV replication (Response Figure 1A and B). Compared with TMEM41B reconstituted cells, in PK-15-overexpression cells, the higher expression level of TMEM41B significantly promoted the replication of TGEV. This may be the reason why the relative expression levels of TMEM41B between TMEM41B-KO-rescue and PK-15-overexpression cells seem to be equivalent, but the TGEV mRNA detected in the RT-qPCR looks to differ by around 4 logs.

To eliminate the impact of low expression efficiency in transiently transfected TMEM41B plasmid, we re-conduct the reconstitution cells experiment. We generate a stable single-clone originated TMEM41B-KO-rescue cell line. As shown in Fig. 2G and H (revised version), the results showed that TGEV replication can be completely rescued in the TMEM41B reconstituted cells. It indicated that TMEM41B is the critical gene for TGEV replication on PK-15 cells.

Response Figure 1

COMMENT:

The above point highlights the lack of information in the Methods section. For example, how did the authors perform the reconstitution experiments? From the Methods it seems that the KO clone constitutively expresses an sgRNA targeting TMEM41B. Did the authors mutate the TMEM41B cDNA so that it is resistant to cleavage? If the TMEM41B cDNA was reconstituted in cells expressing the TMEM41B sgRNA it could explain the partial rescue observed in the TMEM41B-KO-rescue cells.

RESPONSE:

Thank you for helping us improve the clarity of our manuscript. As per your advice, we have added these details for the benefit of readers at lines 1202-1204, as follows, *“**To abolish cleavage by sgRNA and Cas9 in TMEM41B KO cells, a specific point mutation in PAM sequence, which not alter the amino acid was introduced into the TMEM41B coding sequences.”*

The related description of these results has been revised to the manuscript at lines 150-156, as follows: “*To detect whether TMEM41B is required for TGEV infection, we constructed a CRISPR-resistant TMEM41B-KO-rescue cell line, which stably expressed a mutated TMEM41B gene on its KO cells (S2 Fig). As shown in Fig 2G and 2H, the TGEV replication on TMEM41B-KO-rescue cells can restore completely compare with the wild-type (WT) cells. Furthermore, overexpression of TMEM41B in WT cells can promote TGEV replication (Fig 2I and 2J). These results indicated that TMEM41B as a critical gene participate in TGEV replication.*”

Major points (2):

The nomenclature in the manuscript is confusing. The authors use WT and PK-15 and it seems these may be the same but it is unclear. For example, what is the difference between figure 2g and 2i? In supplementary figure 10, overexpression of TMEM41B in PK-15 cells potently inhibits ISG expression compared to PK-15/WT cells, even after treatment with polyI: C. Therefore, lower levels of the ISG transcripts for WT compared to the TMEM41B KO-rescue cells for figure 3f is unexpected. For example, we can see this in supplementary figure 10d–IFIT1 expression is lower in the PK-15-overexpression cells compared to WT in the absence of polyI:C treatment. This should be comparable to IFIT1 in figure 3f, however, higher IFIT1 expression is observed in the TMEM41B-KO-rescue relative to WT. It is impossible to see if this is the case for the rest of the ISGs in figure 10 given the scale of the y-axis.

RESPONSE:

We apologize for this ambiguity. WT and PK-15 in Figures 2g and 2i are the same cells. We have unified the same consistent naming format, which using “WT” in the corresponding Figures.

Thank you for bringing this issue to our attention. As mentioned above, we re-conduct the reconstitution experiment for generation of a stable single-clone originated TMEM41B-KO-rescue cell line (Fig. 2G, revised version) instead of pooled cells to increase the expression level of TMEM41B. Therefore, we re-conduct experiment for detection of ISGs expression in stable TMEM41B reconstituted cells by RT-qPCR assays. As shown in Fig. 3F (revised version), the results showed that the expression level of ISGs in TMEM41B-KO-rescue cells were decreased to the levels of WT cells.

The related description of these results has been revised to the manuscript at lines 196-198, as follows: “*To confirm that the ISG-activation signature was specific to the loss of TMEM41B function, we conducted rescue experiments and found that the mRNA expression of MX1, OASL, IFIT1, IFI6 and RSAD2 in TMEM41B-KO-rescue cells decreased to WT level (Fig 3F).*”

Major points (3):

Figure 6b-f: The striking ISG upregulation observed in the TMEM41B KO cells confounds the replication defect observed for these virus families. The antiviral state in TMEM41B KO cells, beyond the function of TMEM41B, makes it difficult to conclude that the defect is due to the requirement of TMEM41B and not a result of the higher basal ISG levels. The authors address this for TGEV by generating a double KO for TMEM41B and IFNAR, which normalized the ISG expression, and confirm that TGEV replication is still not supported. Therefore, the effects seen for these other viruses could be due to IFN/ISG expression. This is particularly important for understanding the replication defect seen in negative strand-viruses which was not observed in a previous study. It is also mechanistically unclear how negative-strand RNA virus replication would be affected given the proposed role of TMEM41B in replication organelle formation for positive-strand viruses. The inconsistency between their model and how the negative-strand RNA viruses are inhibited is not explained and experiments to address this are not included. These results need to be confirmed in the double KO IFNAR cell lines as done with TGEV. Reconstitution experiments with TMEM41B with these other viral families should also be performed to demonstrate TMEM41B’s role in their replication.

RESPONSE:

Thank you bringing this issue to our attention. As per your suggestion, negative-strand RNA virus, VSV was infected with double KO cell lines to explore mechanism. As shown in Fig. 6G (revised version), VSV titers were increased ~1 log on double KO cells compare to the TMEM41B KO cells. However, we found that double KO cells still have a strong ability to inhibit VSV replication. This result suggested that up-regulation of ISGs mRNA in TMEM41B KO cells only partially inhibit VSV replication. Therefore, we speculate that knocking out TMEM41B may not only inhibit VSV infection by regulating ISGs. The VSV and influenza virus infection requires normal host lipid metabolism [1-3]. Some studies showed that lipid transport is impaired on TMEM41B KO cells [4-6]. It is not clear whether knocking out TMEM41B may inhibit lipid transport and thereby inhibit VSV and influenza virus infection.

The related description of these results has been revised to the manuscript at lines 313-320, as follows: “*Interestingly, we observed that TMEM41B KO led to significantly inhibited replication of the negative-strand RNA virus, PR8 influenza A virus (IAV-PR8, H1N1) (Fig 6E), vesicular stomatitis virus (VSV) (Fig 6F). In addition, TMEM41B KO and TMEM41B/IFNAR double KO and WT cells were infected with VSV, respectively. The results showed that the VSV infectious titer in double KO cells was ~1 log higher than that of TMEM41B KO cells, but it was still significantly lower than that of WT cells (Fig.6G). Therefore, we speculate that knocking out TMEM41B may not only inhibit VSV infection by regulating ISGs.”*

As per your suggestion, to demonstrate the role of TMEM41B in the replication of other virus families, we re-conduct reconstitution experiments and found that rescue expression of TMEM41B in its KO cells can rescue the replication of these virus, such as MHV, PDCoV, JEV, VSV and IAV (Fig. 6B-F, revised version).

The related description of these results has been revised to the manuscript at lines 304-315, as follows: “*Therefore, we examined the potential impacts of TMEM41B on mice MHV infection in TMEM41B KO and its reconstituted L929 cells. Compared with the WT group, the supernatant virus titers of the TMEM41B KO L929 cells were significantly decreased measured in 50% tissue culture toxicity dose assays (TCID50 assay), while the virus replication can be restored on TMEM41B-KO-rescue cells (Fig 6B). In addition, we found that porcine deltacoronavirus (PDCoV) replication was significantly inhibited in TMEM41B KO cells compared with WT cells (Fig 6C) as demonstrated by the reduced supernatant virus titers of the TMEM41B KO cells in the TCID50 assay. Additional assays showed that TMEM41B reduced the replication ability of positive-strand RNA viruses including porcine JEV (Flaviviridae) (Fig 6D). Interestingly, we observed that TMEM41B KO led to* [*significantly*](javascript:;) *inhibited replication of the negative-strand RNA virus, PR8 influenza A virus (IAV-PR8, H1N1) (Fig 6E), vesicular stomatitis virus (VSV) (Fig 6F).*”

Reviewer #2 (Remarks to the Author):

COMMENT:

This thorough submission presents results indicating that TMEM41B renders cells and experimental mice permissive to coronavirus infection. The study follows a standard path, from CRISPR KO screening, to identification and validation of TMEM41B as necessary for robust growth of virus, to focus toward mechanism of TMEM41B operations in virus infection cycles. If there is a central issue, it is that very similar results have been published. The most strikingly similar work was published while this work was submitted, it is and https://doi.org/10.1371/journal.ppat.1009599 and so it is in the PPATH now. However, there may be good arguments for having some duplicated work in the journal. The new findings in this submission are that (1) the TMEM41B suppresses interferon signaling, but this is not the proviral TMEM41B mechanism; (2) the TMEM41B is claimed to facilitate virus endocytosis; (3) the TMEM41B increases C57BL/6 mouse susceptibility to lethal MHV A59 infection. The main findings that TMEM41B is needed for post-entry replicative processes and that TMEM41B facilitates virus replication organelles are also present. These main findings concord with the previous publications on TMEM41B in flavivirus and coronavirus replication. The work is generally solid but there are some comments on the data.

RESPONSE:

We thank the reviewers for pointing out this issue. In the following sections, you will find our responses to each of your points and suggestions.

Major points (1):

Fig. 4d, these data do not provide much confidence in the claim that TMEM41B facilitates virus endocytosis. The figure lacks images of virus particles bound at 4℃ but not yet internalized by shift to 37℃. Given equivalent virus binding to TMEM41B+ and – cells (Fig 4a), one wonders where the viruses are located in TMEM41B-KO cells in Fig 4d.

RESPONSE:

Thank you for pointing out these issues. As per your suggestion, an immunofluorescence assay was performed to further evaluate the adsorption activity of TGEV on TMEM41B KO and WT cells, which were infected with TGEV (MOI = 5) at 4 ℃ for 1 h. As shown in Fig.4B (revised version), we found the virus particles were located on the cell membrane of TMEM41B KO and WT cells. It suggested that viruses bound to the cell membrane of TMEM41B KO cells at 4℃ but not yet internalized by shift to 37℃.

The related description of these results has been revised to the manuscript at lines 219-224, as follows: “*The absorption rate of virus particles in TMEM41B KO and WT cells at 4°C for 1 h was measured. The result showed that these two cell lines had the same absorption capacity of virus particles (Fig 4A). We found that the positive fluorescence signal of TGEV N protein is evenly distributed on the cell membrane of TMEM41B KO and WT cells, indicating that virus particles can still bind to TMEM41B KO cells (Fig 4B). These results suggested that knocking out TMEM41B doesn’t affect the absorption of virus particles by PK-15 cells.*”

Major points (2):

Fig. 5A, it is hard to be sure that the red arrow is pointing to a replication organelle; also, more replication organelles should be imaged to get a better sense of the TMEM41B role in RO formation. Optimally, immunogold EM or similar methods should be used to identify TMEM41B in the ROs.

RESPONSE:

Thanks for this note. We have modified the Fig. 5 as requested. As shown in the enlarged images of TGEV-infected WT cells (Fig.5A and B, revised version), we can clearly observe double membrane vesicles (DMVs). We also observed other types of ROs in the TGEV-infected WT cells, including the small double-membrane spherules (DMSs) and irregular coiled membrane formed by ERs expansion (Fig.5C and D, revised version). In comparison, there was no RO in the TMEM41B KO cells when infected with TGEV (Fig.5E and F, revised version). These results showed that TMEM41B played a critical role in the ROs formation of TGEV.

In addition, we further explored the role of TMEM41B in the formation of the ROs of the SARS-CoV-2. Firstly, we generated a TMEM41B KO HEK293T cell lines. Subsequently, we constructed PLV-mCherry-SARS-CoV-2-NSP3 and PLV-eGFP-SARS-CoV-2-NSP4 lentiviral expression vectors (Supplementary Fig 12A, revised version). After co-infection of PLV-mCherry-SARS-CoV-2-NSP3 and PLV-eGFP-SARS-CoV-2-NSP4 lentivirals in TMEM41B KO and WT HEK293T cells, the double immunofluorescence cells (mCherry and eGFP) were sorted by flow cytometer, then collected and observed by transmission electron microscope. Compared with mock cells, we found that after co-overexpression of NSP3 and NSP4 from SARS-CoV-2 in WT cells, the morphology of ER was visibly swollen and distorted (Supplementary Fig 12A-F, revised version). It was found that many ER membranes have obvious membrane curvatures, resulting in large deformations (Supplementary Fig 12E and F, revised version). The result showed that SARS-CoV-2 NSPs can induce small DMVs with a diameter of ~100 nm in HEK293T cells (Supplementary Fig 12C, revised version), which is consistent with the previous reports of MERS-CoV and SARS-CoV[7]. In contrast, we found that the ER morphology has no obvious swelling or deformation after co-expression of NSP3 and NSP4 in TMEM41B KO cells (Supplementary Fig 12G-L, revised version). These results showed that TMEM41B may play a critical role in the membrane bending during ROs formation of SARS-CoV-2.

A related description of these results has been added to the revised manuscript at lines 256-255 and 280-289.

Major points (3):

Fig. 5bcd, the TMEM41B and TGEV NSP3 proteins are so over-expressed that it is hard to make claims for relevant colocalization. The IFA signals cover most of the cytoplasm. A suggestion is to assess colocalization at lower expression levels (levels consistent with natural expression in infected cells).

RESPONSE:

We sincerely thank you for this kind suggestion. As per your advice, to reduce the protein expression levels of TMEM41B and TGEV NSP3, we performed IFA assay within 18 h after co-transfection with the TMEM41B-FLAG and TGEV-NSP3-HA vectors instead of 24 h in our previous IFA assay. We found that although the protein expression levels of TMEM41B and NSP3 were significantly reduced, they were still found to be co-localized by fluorescence microscopy (Fig 5G, revised version).

Furthermore, we re-conduct assay for analysis of the co-localization of dsRNA and restore expression of TMEM41B-FLAG in its KO cells during TGEV infection via confocal fluorescence microscopy. We found that the fluorescent signal of TMEM41B was significantly accumulated in TMEM41B KO cells infected with TGEV and co-localized with dsRNA (Fig 5H, revised version). DsRNA as the intermediate of CoVs replication and mainly localized to the DMV interior. These results indicated that TMEM41B play an important role in the formation of ROs induced by TGEV.

Reviewer #3 (Remarks to the Author):

COMMENT:

This manuscript reports the identification of TMEM41B as a host factor required for the infection of the transmissible gastroenteritis virus (TGEV), a member of α-coronaviruses. The authors show that TMEM41B is important for the formation of double-membrane vesicles that are derived from the ER and required for viral replication. The authors suggest that TMEM41B also protects mice from coronaviral (MHV) infection. In general, it is important to search for host factors required for viral infections. However, at least six genome-wide CRISPR screening studies (one published in PLOS pathogens) have so far identified TMEM41B as a host factor of coronaviral infection, which reduces the novelty of this study. This study also provides potentially novel findings: (i) the effect of TMEM41B loss on the formation of double-membrane vesicles and (ii) the role of TMEM41B in mice in vivo. Unfortunately, the data of these two findings are not convincing enough in the present version (see specific comments below).

RESPONSE:

We thank the reviewers for pointing out this issue. In the following sections, you will find our responses to each of your points and suggestions.

Major points (1):

The data of knockout mice (Fig. 7) are potentially important but not convincing.

- It is not clear how many mice were used in the experiments in a, b, and f, and what statistical analysis was used in a and b (the t-test is not acceptable). Actual p-values should be shown.

RESPONSE:

We apologize for this ambiguity. In Fig. 7A and B, 6 mice were used in each group; in Fig. 7f, 3 mice were used in each group. The statistical analysis of a two-way ANOVA was used in Fig. 7A, while a one-way ANOVA was used in Fig. 7B, *P* values for each group were provided in Fig. 7A, B and C (revised version). To avoid confusion among readers, we have also included a supplementary explanation in the legend of Fig 7.

COMMENT:

- It is questionable that heterozygous mice indeed have a defect in the function of TMEM41B. Even though heterozygous mice express a reduced amount of TMEM41B, it may be sufficient physiologically. Thus, it is essential to determine whether some of the key functions (e.g., p62 accumulation, autophagic flux, lipid droplet accumulation, coronaviral replication, DMV formation) are indeed defective in cultured TMEM41B^+/-^ cells. Also, the p62 level, lipid droplet accumulation, and serum lipid concentrations should be compared between Wild-type and TMEM41B^+/-^ mice (before infection).

RESPONSE:

Thank you very much for your good question. As per your advice, we conduct the related assays. A related description of these results has been added to the revised manuscript at lines 330-336, as follows: “*Compared with WT mice, we found that the lipidated LC3 II autophagy marker gene levels in the liver and kidney of TMEM41B^+/-^ mice were reduced, indicating that autophagy may be defective in TMEM41B^+/-^ mice (Supplementary Fig 13G, revised version). In addition, the serum lipid concentrations include that triglyceride (TG), total cholesterol, Low-density lipoprotein (LDL) cholesterol in the serum of TMEM41B^+/-^ mice was significantly decreased compared with WT mice (Supplementary Fig 13H, revised version). The results indicated that TMEM41B^+/-^ mice may have defective on lipid transport, which is consistent with previous report.*”

In addition, we established a heterozygous TMEM41B KO L929 cell line using CRISPR/Cas9 and further determine whether it have a defect of coronaviral replication (Response Figure 2). This result showed that heterozygote cell also can reduce MHV replication.

Response Figure 2

COMMENT:

- The authors used 6 mice in the experiment in Panel C. How can there be a 20% reduction?

RESPONSE:

Thank you for catching these errors. We have revised the survival curves on Fig. 7C (revised version).

COMMENT:

- The authors should indicate the generation (F0, F1, or F2 etc.) of the mice used in Fig. 7 and Sup. Fig. 11C-F.

RESPONSE:

We apologize for this ambiguity. The F2 generation of TMEM41B+/- mice were used in Fig. 7 and Supplementary Fig. 13C-F (revised version).

To avoid confusion among readers, we have included a supplementary explanation in the legend of Fig. 7 and Supplementary Fig. 13C-F (revised version).

COMMENT:

- The description "TMEM41B-KO" is inappropriate. This should be "TMEM41B^+/-^".

RESPONSE:

Thank you for all of your thoughtful and constructive advice to strengthen the quality of our manuscript. We are very grateful for your careful consideration. We have unified the formats for "TMEM41B^+/-^" throughout the manuscript.

Major points (2):

The quality of the electron microscopy data is not good. The images are too small to evaluate. In Fig. 5a, "double membranes" are not visible. Images with higher resolution should be presented. In addition, some quantification is required in order to show that TMEM41B is required for the formation of double-membrane vesicles.

RESPONSE:

Thank you for bringing this issue to our attention. As per your suggestion, images with higher resolution of the electron microscope were provided, and even partially enlarged images were displayed (Fig 5A-F, revised version). As shown in Fig 5A and B (revised version), the "double membrane" of DMVs is visible in TGEV-infected WT cells. In addition, the number of DMVs between TMEM41B KO and WT cells were calculated. In the random observation field of 68 different TGEV-infected WT cells by Transmission Electron Microscopy (TEM) assay. In total, we found about 35 DMVs with a diameter of ~200 nm. Our results were consistent with the few DMVs observed in LLC-PK cells infected with PDCoV [8, 9], indicating that it may be related to the same source of porcine kidney passage cells. In contrast, compared with PEDV-infected Vero-E6 cells [10], MHV-infected L929 cells [11] and MERS-CoV-infected Huh7 cells [7], we found that the number of DMVs in TGEV-infected PK-15 cells was relatively less, which makes it difficult for us to quantify the statistical differences of DMVs in KO and WT cells in this study. But obviously, when TGEV infects TMEM41B KO cells, no DMV was found in TMEM41B KO cells (Fig 5F, revised version). These results indicated that TMEM41B is required for the formation of DMVs.

Interestingly, we found that there are many vesicles were present in TMEM41B KO cells (Fig 5E and F, revised version). Different from virus-induced organelles, the vesicles were monolayer and different morphologies with varying sizes. These unidentified vesicles were similar to the previously reported features, which maybe the precursor of the autophagosome that originated from ER membrane [12]. The latest studies reported that TMEM41B as a phospholipid scramblase mediating trans-bilayer shuttling of phospholipids [13-15]. Combined with our newly discovered results, we provide a hypothetical model for the role of TMEM41B involved in the TGEV replication (Fig 5J, revised version). In WT cells, TMEM41B may mediate the phospholipids synthesized on the cytoplasmic lobules, and shuttle to the luminal lobules to promote the balance of the phospholipid bilayer. When cells are infected by a coronavirus, the transmembrane proteins of NSPs (NSP3, NSP4 and NSP6) encoded by the coronaviruses located in the endoplasmic reticulum will oligomerize, and recruit TMEM41B to increase membrane curvature, thereby leads to the formation of DMVs. When the cells lack of TMEM41B, the NSPs encoded by the coronavirus will not be able to generate DMVs by changing the membrane curvature of the endoplasmic reticulum.

A related description of these results has been added to the revised manuscript at lines, 256-265 and 290-299.

Major points (3):

It is not clear how the authors distinguish between viral internalization and replication. They use essentially the same method at different time points. This reviewer is not sure whether this is sufficient.

RESPONSE:

Thank you for raising this question. It is reported that after the adsorption of coronavirus infection, it will be quickly internalized by host cells [16]. Confocal microscopy can directly observe the early events of coronavirus infection. This is achieved by detecting the expression of TGEV N protein to trace the entry process of the coronavirus genome [17]. In our study, PK-15 cells were infected with high virus titer TGEV (MOI=50) for 30 min at 37°C, we found that the fluorescence signal of TGEV N protein mainly located on the cytoplasm of WT cells (Fig 4C, revised version). The results indicated that the internalized TGEV infection stage can be detected under this condition.

To determine the early stage of TGEV replication in PK-15 cells, IFA assays were performed to detect the expression of N protein in TGEV-infected cells at 1, 2 and 3 h.p.i. As shown in Response Figure 3, we found that after 3 h of infection with TGEV (MOI=5), TGEV N protein began to be expressed largely in PK-15 cells, but there was no obvious positive fluorescence signal at 1 or 2 h.p.i. It can be seen that the early replication of TGEV may start at ~3 h.p.i. Therefore, the time point 3 h after virus infection was selected to study the effect of TMEM41B on the early stage of TGEV replication, thereby distinguishing the internalization and replication stages (Fig. 4G and H, revised version).

Response Figure 3

**Minor Issues:**

COMMENT:

Please use this section for editorial suggestions as well as relatively minor modifications of existing data that would enhance clarity.

Reviewer #1:

(1) Lines 119-120: “Our screen found that a total of 335 unique sgRNA sequences, which targeting to 317 unique protein-coding genes” There seems to be no convergence of sgRNAs per gene identified in the screen – essentially one guide per gene. One would expect that with multiple sgRNAs targeting a single gene, there should be multiple sgRNAs enriched for each of the hits. This weakens the reliability of the screen data. Was the screen performed with replicates? At what fold-representation was the screen performed? There is no information about the full-genome screen in the Methods section.

RESPONSE:

Thank you for this constructive comment. Among the top 0.1% of sgRNAs, multiple sgRNAs targeting the same gene were identified. For example, in the second challenge rounds, 2 and 3 sgRNAs hit the ANPEP and TMEM41B genes, respectively (S1 Dataset, revised version). Moreover, a focused CRISPR library screening was performed, even 5 rounds of TGEV (MOI=1) repeated challenge assays were conducted. We found that 4 sgRNAs hit TMEM41B in the focused CRISPR library screening datasets (Fig. 2A). These results showed that through multiple rounds of repeated whole genome-wide and focused CRISPR screening, the hit targets found are reliable.

We conducted three rounds of TGEV challenge instead of replicates. In the screen, PigGeCKO cells were generated by a total of ~2 × 10^8^ PK-15-Cas9 cells and infected with the genome-wide sgRNA library lentiviruses at an MOI of 0.3. Thus, the number of cells reach about 700-fold for total sgRNAs in the library. Thus, the screen was performed at ~700-fold representation. The methods of whole genome-wide screening as described in our previous publication (“CRISPR screening of porcine sgRNA library identified host factors facilitating Japanese encephalitis virus replication”). And we have described this in the Methods section, as follows: “The PigGeCKO cells were generated by a total of ~2 × 10^8^ PK-15-Cas9 cells and infected with the genome-wide sgRNA library lentiviruses at an MOI of 0.3 as described in a previous publication[18]”.

(2) Based on the results presented, TMEM41B was not the most reliable hit from the screens. What was the rationale for choosing TMEM41B as opposed to the other genes identified as top hits in the screen?

RESPONSE:

Thank you for raising this question. To identify host factors involved in α-CoV TGEV replication, a genome-wide loss-of-function genetic screen was first performed. We conducted three rounds of TGEV (MOI=0.001) challenge. As shown in Fig.1C, we found that TMEM41B ranked in the top 10 candidate genes list. Next, we performed a focused-CRISPR library screening which examined the 79 top-ranked candidate genes from the genome-wide KO screen, note that we did not include any sgRNAs targeting the previously-identified ANPEP receptor. We conducted five rounds of TGEV (MOI=1) challenge. As shown in Fig. 2A, we still found that TMEM41B ranked in the top 10 candidate genes list, which indicated that knocking out TMEM41B on PK-15 cells can inhibit TGEV infection at high MOI levels. In addition, we found that the ability of knocking out TMEM41B and ANPEP receptors to inhibit TGEV infection is very close (Fig 2B and C). Therefore, TMEM41B was chosen as it is the most reliable hit from the screens.

(3) Figure 2f-j: What time point were the cells harvested at?

RESPONSE:

Thank you for raising this question. In Fig.2F, we harvested cells 24 h after TGEV infection. In Fig.2G and J, we harvest the cells 12 h after TGEV infection.

(4) Figure 2g-j: What is the difference between WT-NTC and PK-15-NTC? If these are the same consistent naming would helpful.

RESPONSE:

We apologize for this ambiguity. WT-NTC and PK-15-NTC in Figures 2g and 2i show the same treatment. We have unified the same consistent naming format, which using “WT-NTC” in the corresponding Figures.

(5) Figure 3e and f: why are there such large discrepancies between the relative fold change between figures e and f? For example, there is 100-fold difference for MX1 when comparing WT and TMEM41B KO in figure 3e but only a 10-fold difference for MX1 in figure 3f. Would be nice to have an idea of the raw transcript numbers for these ISGs and see how this may influence the large fold changes observed in these experiments.

RESPONSE:

Thank you for pointing out these issues. Although Fig. 3E and F showed the results of RT-qPCR for the same gene, such as MX1, OASL, but the samples weren't the same cells group, this maybe the reason for the results showed that discrepancies between the relative fold change between Fig. 3E and F. In addition, the raw transcript numbers for these ISGs were found that: MX1: 4584.680721; OASL: 162.3795295; IFIT1: 7663.500127; IFI6: 594.6129877; RSAD2: 23792.3155. And these results can be found in the Supplementary Data file 3.

(6) Figure 3: are there differences in IFN mRNA levels between the various cell lines? Does this change if TMEM41B is overexpressed?

RESPONSE:

Thank you for these suggests. As per your suggestion, we detected that IFN mRNA levels of the WT, TMEM41B-KO and TMEM41B-KO-rescue cells. As shown in Response Figure 4, we found that knock out of TMEM41B can up-regulation IFN α and β mRNA levels in PK-15 cells. In addition, restoring TMEM41B on its KO cells can reduce IFN α and β mRNA levels close to WT levels.

Response Figure 4

(7) Figure 3: Other cell lines should be tested to see the ISG upregulation observed in the TMEM41B KO cells is cell line specific. This would help to explain why their results differ from those previously reported.

RESPONSE:

Thank you for these suggests. As per your suggestion, we generation of TMEM41B KO human HEK293T cells and mouse L929 cells by CRISPR/Cas9 technology (Supplementary Fig10A and C, revised version). Compared with TMEM41B KO PK-15 cells, we found that knocking out TMEM41B only slightly increased the expression of ISGs in HEK293T and L929 cells (Supplementary Fig10, revised version), indicating that the ability to up-regulate ISGs varies by cell type.

A related description of these results has been added to the revised manuscript at lines 198-202, as follows, “*In addition, TMEM41B KO human HEK293T cells and mouse L929 cells were generated by CRISPR/Cas9 technology (S10A and S10C Fig). Compared with TMEM41B KO PK-15 cells, we found that knocking out TMEM41B only slightly increased the expression of ISGs in HEK293T and L929 cells, indicating that the ability to up-regulate ISGs varies by cell type (S10B and S10D Fig).”*

(8) Supplementary figure 10: It is surprising that the PK-15 cells overexpressing TMEM41B completely inhibits the upregulation of ISGs when treated with polyI:C. Further work to show if this is polyI:C specific or to help explain these results should be demonstrated.

RESPONSE:

Thank you for pointing out these issues. Here, we performed SEV-stimulated Dual-luciferase assay to detect whether TMEM41B inhibits the upregulation of ISGs for polyI:C is specific. In brief, the cells were seeded into 24-well plates were co-transfected with a luciferase reporter plasmid (ISREs-Luc, interferon stimulation reaction element-Luc) and pRL-TK plasmid (Promega, WI, USA), along with the porcine TMEM41B plasmids or empty vector for 24 h, and were then infected with SeV (10 hemagglutinating activity units/well). At 10 h post-infection, the cells were lysed, and were detected through the Dual Luciferase Reporter Assay Kit (Vazyme, DL101-1) according to the protocol. Representative data from three independently conducted experiments are shown as the relative firefly luciferase activities with normalization to the Renilla luciferase activities. The result showed that showed that overexpression of TMEM41B significantly blocked the ISREs stimulated by Sendai virus (SeV) in HEK293T cells (Response Figure 5). Therefore, we suggested that overexpression of TMEM41B may inhibit the upregulation of ISGs is not only for polyI:C specific.

Response Figure 5

(9) Figure 4c: The low image resolution/magnification makes it impossible to state that no DMV formation is observed in the TMEM41B KO cells. We can see the absence of viral particles in the TMEM41B KO cells compared to the WT, but it cannot be determined from this image if DMVs are present.

RESPONSE:

Thank you for pointing out these issues. As mentioned above, we have modified the Fig. 4 and 5 (revised version) as requested. As shown in the enlarged images of TGEV-infected WT cells (Fig.5A and B, revised version), we can clearly observe double membrane vesicles (DMVs). We also observed other types of replication organelles in the TGEV-infected WT cells, including the small open double-membrane spherules (DMSs) and irregular coiled membrane formed by ERs expansion (Fig.5C and D, revised version).

(10) Figure 4: The authors suggest viral endocytosis may contribute to the replication defect observed. It has been shown in other studies that when viral RNA is directly transfected into cells (bypassing endocytosis) a replication defect is still observed in TMEM41B KO cells. This highlights the need for Sun et al to compare their results to previous reports that used different viruses and host species and discuss the similarities and differences.

RESPONSE:

Thank you for this valuable suggestion. In this study, we first reported the importance of TMEM41B in the endocytosis of CoVs. There are some studies reported that TMEM41B is critical for mediating lipid mobilization, TMEM41B KO cells have apparent accumulation of free cholesterol[4-6]. Meanwhile, cholesterol allowing membrane rearrangements that facilitate TGEV entry and is important for a post-adsorption step[19]. Therefore, we suggested that impaired endocytosis of TMEM41B KO cells maybe relate with lipid mobilization defect. Since we only use TGEV-infected PK-15 cells as a model to study the endocytosis process, it is impossible to predict whether this phenomenon is the same as other viruses and hosts.

Our results suggested that TMEM41B KO cells inhibit the formation of ROs induced by TGEV (Fig. 5E and F, revised version), we found knockout TMEM41B almost completely inhibited the early replication of TGEV (Fig. 4G and H, revised version), which was consistent with previous reports [20, 21]. In addition, we also found that SARS-CoV-NSP3 and NSP4 could not induce the formation of DMVs in TMEM41B KO cells, Therefore, we believed that that TMEM41B plays a conservative role in the formation of CoVs ROs.

**Reviewer #2:**

(1) Fig. 1; consider highlighting the TMEM41B in panel b and d scatter plots

RESPONSE:

We sincerely thank you for this kind suggestion. we have modified the Fig. 1 as requested.

(2) Fig. 4b, hard to be sure that the red arrows are pointing to virus particles (but evidence for less virus morphogenesis in TMEM41B-KO is strong from other results)

RESPONSE:

Thank you for pointing out these issues. As per your suggestion, we have modified the Fig. 4, the images with higher resolution of the electron microscope were provided. As shown as Fig. 4E, (revised version), there are large virion-containing vacuoles (LVCVs) [9] were observed in WT cells (Fig. 4E, revised version).

(3) The text has many confusing sentences, too many to itemize. Some examples include lines 94-100; this section summarizes results but does so in an awkward way. Authors would benefit from rephrasing this part. Lines 160-161; VTT domain is not in TGEV, it is in the TMEM41B related proteins. Rephrase this. Lines 200-202, requires rephrasing. Lines 221-222, this sentence is actually misleading and does not summarize the actual result, rephrase to emphasize that ratios of extra vs intra-cellular viral PFU are being assessed. Lines 374-375; requires rephrasing. There are more issues and there should be another go at revising the text.

RESPONSE:

Thank you for helping us improve the clarity of our manuscript. As per your advice, we carefully corrected all of these kinds of errors in revised manuscript.

-The sentence in lines 94-100 (lines 89-93, revised version) was revised as “*Our results revealed that KO of TMEM41B inhibits the internalization and early-stage replication of CoV. Importantly, we found that TMEM41B contributes to the formation of CoV replication organelles (ROs). Our studies emphasize the significance of TMEM41B for CoV infection in vivo and in vitro, which indicates that TMEM41B might be a vulnerable target for the development of anti-CoV therapeutics.*”

-The sentence in lines 160-161 (lines 159-160, revised version) was revised as “*Subsequently, the role of other TMEM41B homologous proteins with a VTT domain was determined, such as TMEM41A and vacuole membrane protein 1 (VMP1).*”

-The sentence in lines 200-202 (lines 196-198, revised version) was revised as “*To confirm that the ISG-activation signature was specific to the loss of TMEM41B function, we conducted rescue experiments and found that the mRNA expression of MX1, OASL, IFIT1, IFI6 and RSAD2 in TMEM41B-KO-rescue cells decreased to WT level (Fig 3F).*”

-The sentence in lines 221-222 (lines 238-240, revised version) was revised as “*Compared with WT cells, the extracellular and intracellular viral titers of TMEM41B KO cells were roughly equally, both lower than WT cells (Fig 4F).*”

-The sentence in lines 374-375 (lines 403-404, revised version) was revised as “*Here, we found that TMEM41B plays an important role in the formation of DMV, which may improve the maturation of DMV by increasing the curvature of the ER membrane.*”

(4) The discussion section does not make any speculations on the roles that TMEM41B might have during minus strand RNA virus and DNA virus replication. Instead, there are only the statements on lines 388-390. There is an unrealized opportunity here.

RESPONSE:

Thank you for pointing out these issues. These related discussions have been added to the revised manuscript at lines 419-422, as follows: “*Perhaps the most striking finding from our study is that TMEM41B KO cells displayed broad-spectrum antiviral effects on other viruses, including the positive-strand RNA virus JEV, the negative-strand RNA viruses IAV and VSV. We found that TMEM41B and IFNAR double KO cells still have a strong ability to inhibit VSV replication. Therefore, we speculate that knocking out TMEM41B not only inhibit VSV infection by regulating ISGs. The VSV and influenza virus infection requires normal host lipid metabolism [69-71]. Some studies showed that lipid transport is impaired on TMEM41B KO cells [36, 60, 63]. It is not clear whether knocking out TMEM41B may inhibit lipid transport and thereby inhibit VSV and influenza virus infection.*”

**Reviewer #3:**

(1) The recently identified role of TMEM41B as a lipid scramblase should be included in Discussion. (doi: 10.1073/pnas.2101562118, doi: 10.1083/jcb.202103105, doi: 10.1016/j.cmet.2021.05.006)

RESPONSE:

We sincerely thank you for this kind suggestion. We agree that the roles of TMEM41B as a lipid scramblase is very useful information [13-15], which can help us explore its role in TGEV replication, especially for the formation of DMVs (Fig 5J, revised version). These recent studies have already included in Results and Discussion.

These related discussions have been added to the revised manuscript at lines 409-412, as follows, “*The latest studies reported that TMEM41B as a phospholipid scramblase mediating trans-bilayer shuttling of phospholipids [13-15], which can help us explore its role in CoVs replication, especially for the formation of DMVs.*”

(2) Sequence IDs and alignment methods should be described in the legends or Methods.

RESPONSE:

Thank you for pointing out these issues. As per your suggestion, the sequence IDs and alignment methods were described in the legends of Fig. 6A (revised version), lines 1138-1142, as follows: *“**The accession Numbers of TMEM41B from NCBI protein database for Sus scrofa is XP_003129438.1 (pTMEM41B), for Homo sapiens is NP_055827.1 (hTMEM41B), and for Mus musculus is NP_705745.3 (mTMEM41B). Protein sequences were aligned using Constraint-based Multiple Alignment Tool (COBALT).”*

(3) Supplementary Fig. 5: Small but significant amounts of LC3-II are observed in ATG5-KO and ATG7-KO cells. These are not typical results, raising a concern that isolation of KO cells was not appropriate. Please carefully check them. Otherwise, the authors' claim that "the canonical autophagy pathway is not required for TGEV replication" (L184-185) is not valid.

RESPONSE:

Thank you bringing this issue to our attention. As per your suggestion, we reconstructed an ATG5 KO cell line and determined whether it affects TGEV replication. As shown in Fig. 5 (revised version), we found that the activity of autophagy pathway of ATG5 KO cells was inhibited, but compared with WT cells, the replication ability of TGEV was not reduced.

References:

1.	Wang W, Fu YJ, Zu YG, Wu N, Reichling J, Efferth T. Lipid rafts play an important role in the vesicular stomatitis virus life cycle. Arch Virol. 2009;154(4):595-600. Epub 2009/03/17. doi: 10.1007/s00705-009-0348-2. PubMed PMID: 19288237.

2.	Haldar S, Mekhedov E, McCormick CD, Blank PS, Zimmerberg J. Lipid-dependence of target membrane stability during influenza viral fusion. J Cell Sci. 2018;132(4). Epub 2018/07/04. doi: 10.1242/jcs.218321. PubMed PMID: 29967032; PubMed Central PMCID: PMCPMC6398481.

3.	Elkins MR, Williams JK, Gelenter MD, Dai P, Kwon B, Sergeyev IV, et al. Cholesterol-binding site of the influenza M2 protein in lipid bilayers from solid-state NMR. Proc Natl Acad Sci U S A. 2017;114(49):12946-51. Epub 2017/11/22. doi: 10.1073/pnas.1715127114. PubMed PMID: 29158386; PubMed Central PMCID: PMCPMC5724280.

4.	Kang ZB, Moutsatsos I, Moretti F, Bergman P, Zhang X, Nyfeler B, et al. Fluopack screening platform for unbiased cellular phenotype profiling. Sci Rep. 2020;10(1):2097. Epub 2020/02/09. doi: 10.1038/s41598-020-58861-3. PubMed PMID: 32034186; PubMed Central PMCID: PMCPMC7005823.

5.	Moretti F, Bergman P, Dodgson S, Marcellin D, Claerr I, Goodwin JM, et al. TMEM41B is a novel regulator of autophagy and lipid mobilization. EMBO Rep. 2018;19(9). Epub 2018/08/22. doi: 10.15252/embr.201845889. PubMed PMID: 30126924; PubMed Central PMCID: PMCPMC6123663.

6.	Trimarco JD, Heaton BE, Chaparian RR, Burke KN, Binder RA, Gray GC, et al. TMEM41B is a host factor required for the replication of diverse coronaviruses including SARS-CoV-2. PLoS Pathog. 2021;17(5):e1009599. Epub 2021/05/28. doi: 10.1371/journal.ppat.1009599. PubMed PMID: 34043740; PubMed Central PMCID: PMCPMC8189496.

7.	Oudshoorn D, Rijs K, Limpens R, Groen K, Koster AJ, Snijder EJ, et al. Expression and Cleavage of Middle East Respiratory Syndrome Coronavirus nsp3-4 Polyprotein Induce the Formation of Double-Membrane Vesicles That Mimic Those Associated with Coronaviral RNA Replication. mBio. 2017;8(6). Epub 2017/11/23. doi: 10.1128/mBio.01658-17. PubMed PMID: 29162711; PubMed Central PMCID: PMCPMC5698553.

8.	Doyle N, Hawes PC, Simpson J, Adams LH, Maier HJ. The Porcine Deltacoronavirus Replication Organelle Comprises Double-Membrane Vesicles and Zippered Endoplasmic Reticulum with Double-Membrane Spherules. Viruses. 2019;11(11). Epub 2019/11/07. doi: 10.3390/v11111030. PubMed PMID: 31694296; PubMed Central PMCID: PMCPMC6893519.

9.	Qin P, Du EZ, Luo WT, Yang YL, Zhang YQ, Wang B, et al. Characteristics of the Life Cycle of Porcine Deltacoronavirus (PDCoV) In Vitro: Replication Kinetics, Cellular Ultrastructure and Virion Morphology, and Evidence of Inducing Autophagy. Viruses. 2019;11(5). Epub 2019/05/22. doi: 10.3390/v11050455. PubMed PMID: 31109068; PubMed Central PMCID: PMCPMC6563515.

10.	Zhou X, Cong Y, Veenendaal T, Klumperman J, Shi D, Mari M, et al. Ultrastructural Characterization of Membrane Rearrangements Induced by Porcine Epidemic Diarrhea Virus Infection. Viruses. 2017;9(9). Epub 2017/09/06. doi: 10.3390/v9090251. PubMed PMID: 28872588; PubMed Central PMCID: PMCPMC5618017.

11.	Gosert R, Kanjanahaluethai A, Egger D, Bienz K, Baker SC. RNA replication of mouse hepatitis virus takes place at double-membrane vesicles. J Virol. 2002;76(8):3697-708. Epub 2002/03/22. doi: 10.1128/jvi.76.8.3697-3708.2002. PubMed PMID: 11907209; PubMed Central PMCID: PMCPMC136101.

12.	Morita K, Hama Y, Izume T, Tamura N, Ueno T, Yamashita Y, et al. Genome-wide CRISPR screen identifies TMEM41B as a gene required for autophagosome formation. J Cell Biol. 2018;217(11):3817-28. Epub 2018/08/11. doi: 10.1083/jcb.201804132. PubMed PMID: 30093494; PubMed Central PMCID: PMCPMC6219718.

13.	Huang D, Xu B, Liu L, Wu L, Zhu Y, Ghanbarpour A, et al. TMEM41B acts as an ER scramblase required for lipoprotein biogenesis and lipid homeostasis. Cell Metab. 2021;33(8):1655-70 e8. Epub 2021/05/21. doi: 10.1016/j.cmet.2021.05.006. PubMed PMID: 34015269.

14.	Li YE, Wang Y, Du X, Zhang T, Mak HY, Hancock SE, et al. TMEM41B and VMP1 are scramblases and regulate the distribution of cholesterol and phosphatidylserine. J Cell Biol. 2021;220(6). Epub 2021/05/01. doi: 10.1083/jcb.202103105. PubMed PMID: 33929485; PubMed Central PMCID: PMCPMC8077175.

15.	Ghanbarpour A, Valverde DP, Melia TJ, Reinisch KM. A model for a partnership of lipid transfer proteins and scramblases in membrane expansion and organelle biogenesis. Proc Natl Acad Sci U S A. 2021;118(16). Epub 2021/04/15. doi: 10.1073/pnas.2101562118. PubMed PMID: 33850023; PubMed Central PMCID: PMCPMC8072408.

16.	Wang J, Li Y, Wang S, Liu F. Dynamics of transmissible gastroenteritis virus internalization unraveled by single-virus tracking in live cells. FASEB J. 2020;34(3):4653-69. Epub 2020/02/06. doi: 10.1096/fj.201902455R. PubMed PMID: 32017270; PubMed Central PMCID: PMCPMC7163995.

17.	Owczarek K, Szczepanski A, Milewska A, Baster Z, Rajfur Z, Sarna M, et al. Early events during human coronavirus OC43 entry to the cell. Sci Rep. 2018;8(1):7124. Epub 2018/05/10. doi: 10.1038/s41598-018-25640-0. PubMed PMID: 29740099; PubMed Central PMCID: PMCPMC5940804.

18.	Zhao C, Liu H, Xiao T, Wang Z, Nie X, Li X, et al. CRISPR screening of porcine sgRNA library identifies host factors associated with Japanese encephalitis virus replication. Nat Commun. 2020;11(1):5178. Epub 2020/10/16. doi: 10.1038/s41467-020-18936-1. PubMed PMID: 33057066; PubMed Central PMCID: PMCPMC7560704.

19.	Yin J, Glende J, Schwegmann-Wessels C, Enjuanes L, Herrler G, Ren X. Cholesterol is important for a post-adsorption step in the entry process of transmissible gastroenteritis virus. Antiviral Res. 2010;88(3):311-6. Epub 2010/10/19. doi: 10.1016/j.antiviral.2010.10.002. PubMed PMID: 20951168; PubMed Central PMCID: PMCPMC7114218.

20.	Schneider WM, Luna JM, Hoffmann HH, Sanchez-Rivera FJ, Leal AA, Ashbrook AW, et al. Genome-Scale Identification of SARS-CoV-2 and Pan-coronavirus Host Factor Networks. Cell. 2021;184(1):120-32 e14. Epub 2021/01/01. doi: 10.1016/j.cell.2020.12.006. PubMed PMID: 33382968; PubMed Central PMCID: PMCPMC7796900.

21.	Hoffmann HH, Schneider WM, Rozen-Gagnon K, Miles LA, Schuster F, Razooky B, et al. TMEM41B Is a Pan-flavivirus Host Factor. Cell. 2021;184(1):133-48 e20. Epub 2020/12/19. doi: 10.1016/j.cell.2020.12.005. PubMed PMID: 33338421; PubMed Central PMCID: PMCPMC7954666.
