# Supplementary material for: Genome-scale CRISPR screen identifies TMEM41B as a multi-function host factor required for coronavirus replication
Source: PLoS Pathog. 2021 Dec 6;17(12):e1010113. doi: 10.1371/journal.ppat.1010113 (PMC8675922; doi:10.1371/journal.ppat.1010113)
Supplement: Attachment — Submitted filename: response to reviewers .docx [file ppat.1010113.s019.docx]

**Point-by-point Response to the reviewers' comments**

**Editor:**

Thank you very much for submitting your manuscript "Genome-scale CRISPR screen identifies TMEM41B as a multi-function host factor required for coronavirus replication" for consideration at PLOS Pathogens. As with all papers reviewed by the journal, your manuscript was reviewed by members of the editorial board and by several independent reviewers. The reviewers appreciated the attention to an important topic. Based on the reviews, we are likely to accept this manuscript for publication, providing that you modify the manuscript according to the review recommendations.

*RESPONSE:*

Thank you for your kind comments on our manuscript entitled “*Genome-scale CRISPR screen identifies TMEM41B as a multi-function host factor required for coronavirus replication*”. We have carefully revised the manuscript according to the reviewer’s comments. Based on the suggestions, we have made an extensive modification on the revised manuscript. Detailed revision was shown as follows. The changes to our manuscript within the document were also highlighted by using red colored text.

**Editor:**

Requests for data to be shown in the actual manuscript or supplementary files, instead of just mentioned in text or only shown in the response to the review are justified and should be fixed.

*RESPONSE:*

According to the reviewer’s comments, we have revised the manuscript extensively and fixed the related data to be shown in the actual manuscript or supplementary files instead of just mentioned in text or only shown in the response to the reviews.

**Reviewers**

Reviewer #2: The authors responded strongly to previous reviews by providing additional results that significantly reinforce the key conclusions of the paper. They complemented TMEM41B KO cells with TMEM41B genes, did this properly, and this added important results demonstrating TMEM41B as a central virus permissively factor. They added new results that now show virus endocytosis and virus-induced DMVs at higher resolution, thereby supporting the contention that TMEM41B operates to support entry and DMV formation. They nicely illustrated distinct functions for TMEM41B in innate immune response, virus endocytosis, virus replication organelle formation, and in vivo virus infection in the liver. They included procedural details that now make it possible for readers to fully appreciate the experiments and results. They appropriately extended discussion of the findings and their contributions to the field.

RESPONSE:

Thank you again for your positive comments and valuable suggestions to improve the quality of our manuscript.

Reviewer #3: The authors addressed most of my previous comments, but there remain some issues to be addressed.

RESPONSE:

Thanks. We have corrected these mistakes based on your suggestions.

Reviewer #4: The authors address many of the reviewer concerns in the revised manuscript. As before, the strengths of this manuscript include the in vivo work and the novel innate immune phenotype observed in the TMEM41B knockout (KO) PK-15 cells. The additional results with TMEM41B/IFNAR double KO PK-15 cells supports the role of TMEM41B in suppressing innate immune activation in PK-15 cells; however, as the authors point out, the effect is minor in other cell types. Indeed, the results in HEK293T and L929 cells are less convincing without additional controls. The ISG upregulation in TMEM41B KO cells may therefore not be generalizable to other cell types. Further, there is a missed opportunity to test other viruses in the TMEM41B/IFNAR1 double KO cells to rule out the possibility that reduced virus replication in TMEM41B KO cells results from elevated ISGs. Nevertheless, the innate immune signature in TMEM41B KO PK-15 cells will be of interest to the field and is worthy of further investigation. In the Discussion section, the authors may wish to speculate more on possible mechanisms by which TMEM41B regulates innate immunity.

RESPONSE:

Thanks for your nice suggestions. According to your suggestions, we have added necessary discussion and edited our article extensively.

The related discussion about possible mechanisms by which TMEM41B regulates innate immunity has been added to the revised manuscript at lines 420-427, as follows: *“In addition, autophagy was found to be a homeostatic process with several effects on immunity [63]. Autophagy represents an anti-inflammatory mechanism in its engagement with inflammasomes, and their substrates protect against endomembrane damage triggered by various agents of endogenous or infectious origin and prevent unnecessary or excessive inflammation [64, 65]. The absence of autophagy may also amplify DDX58/RIG-I-like receptor signaling through increased mitochondrial antiviral signaling protein levels on accumulating mitochondria [66]. Therefore, the impaired autophagy pathway on TMEM41B KO cells may trigger excessive innate immunity.”*

Comments on revised manuscript

Major point 1:

The authors addressed the TMEM41B-KO-resuce by generating a stable single-clone cell line reconstituted with WT TMEM41B. They also include more detail on the methods used to generate the cell line. TMEM41B is an intrinsic regulator of innate immunity. The authors also test human HEK293T and mouse L929 cell lines to determine if the innate immune regulation is observed in other cell types. These data are questionable without additional controls. The gene expression differences are minor and can easily be explained by clone-to-clone variability. As a result, it remains unclear if TMEM41B’s role in suppressing innate immunity is specific to PK-15 cells. Nevertheless, experiments conducted in this manuscript support the author’s claim of innate immune regulation by TMEM41B in PK-15 cells.

RESPONSE:

Thank you for raising this question. Compared with HEK293T and L929 cells, we believe that the role of TMEM41B in suppressing innate immunity is specific to PK-15 cells. To avoid confusion among readers, we have corrected the legend of Fig. S10 *“Knockout of TMEM41B in HEK293T and L929 cells up-regulated the expression of ISGs.”* into *“Evaluation of the ability of TMEM41B to regulate the expression of ISGs in different types of cells.”*

Major point 3:

The authors addressed the concern for figure 6B-G by testing the viruses in TMEM41B reconstituted cells and testing VSV in the TMEM41B/IFNAR double KO cell line. It would be nice to have conducted the TMEM41B reconstitution experiment in the TMEM41B/IFNAR double KO cell line since WT is not the best comparison after generating and growing out single cell clones (particularly after multiple rounds as would have been done for the double KO cell line). Again, there seems to be some missed opportunity here as the innate immune phenotype is one of the more interesting findings in the study. It leaves the reader to wonder how IAV infects the double KO cell line.

RESPONSE:

We thank the reviewers for pointing out this issue. According to your suggestion, we conducted a TMEM41B reconstitution experiment in the TMEM41B/IFNAR double KO cells to evaluate the impact on the replication ability of VSV and IAV. We found that VSV titers were increased ~1 log on double KO cells compare to the TMEM41B KO cells (Fig. 6G, revised version). However, we found that rescue TMEM41B in the TMEM41B/IFNAR double KO cells further increase VSV replication compare to the TMEM41B/IFNAR double KO cells. This result showed that upregulation of ISGs levels in TMEM41B KO cells may only partially inhibit VSV replication, suggesting that TMEM41B may participate in VSV replication through other mechanisms. In comparison, the replication of IAV increase significantly on TMEM41B/IFNAR double KO cells compares to the TMEM41B KO cells (Fig. 6H, revised version). In addition, rescue TMEM41B in the TMEM41B/IFNAR double KO cells does not further facilitate IAV replication. This result suggested that upregulation of ISGs levels in TMEM41B KO cells may be the main factor inhibiting IAV replication. According to your nice suggestions, we have made extensive corrections to our previous draft.

**Major Issues: Key Experiments Required for Acceptance**

Reviewer #2: The opinion of this reviewer is that major issues have now been resolved and key experiments required for acceptance have now been completed.

RESPONSE:

We would like to thank you first for all the positive comments of our manuscript. We really appreciate your help and patience.

Reviewer #3 Previous Major Point 1：

The authors now show that the levels of serum lipids and tissue LC3-II are reduced (Fig. S13). Although the quantitative data of serum TG and LDL cholesterol levels are convincing, the authors’ claim that autophagic activity is reduced is not supported by these data. It has been shown that LC3-II rather accumulates in TMEM41B-/- cells (Ref #34-36). Therefore, the reduction of LC3-II in TMEM41B+/- tissues does not indicate autophagy suppression. Rather, it is actually the opposite. The authors have not included any other autophagic flux data (e.g., p62, LC3 turnover, GFP-RFP-LC3, etc.). Without such data, it would be fair to state that “while serum lipid levels are reduced, autophagic activity may not be affected in heterozygous mice”.

RESPONSE:

Thank you for bringing this issue to our attention. To further determine whether heterozygous mice are autophagy-deficient, after 12 hours of starvation, the p62 protein levels of wild-type and TMEM41B^+/-^ mice were compared. We found that the impairment of autophagic flux characterized by p62 accumulation was significantly increased in the liver and kidney of TMEM41B^+/-^ mice compared with that in WT mice (Fig. S13G, revised version). This result suggested that autophagic activity was disturbed in TMEM41B^+/-^ mice. According to your nice suggestions, we have made extensive corrections to our previous draft.

The information of the number of mice and the method of statistics is still lacking in the legend to Fig. 7A, B and F. Please include.

RESPONSE:

We were really sorry for our careless mistakes. Thank you for your reminding. The missing information has been already added (Fig. 7A, B and F, revised version).

Previous Major Point 2:
The results of the quantification of DMVs should be shown in the manuscript, not only in the rebuttal letter.

RESPONSE:

I very much appreciate the time and effort you’ve put into your comments. As per your suggestion, the related description of these results has been added to the revised manuscript at lines 270-272, as follows: *“The number of DMVs between TMEM41B KO and WT cells were calculated. In the 68 random observation fields of TGEV-infected WT cells by TEM assay, ~35 DMVs with a diameter of ~200 nm were found.”*

**Minor Issues: Editorial and Data Presentation Modifications**

Reviewer #2:

1. The authors have done a good job at determining whether TMEM41B confers cell susceptibility (virus entry, Figs 4AF) or cell permissively (virus replication, Fig 4GH and others). While they have not fully quantified the relative TMEM41B support for each step, the data point strongly to the replication stage being the one that is most dependent on TMEM41B, and likely the most biologically relevant stage. This could be emphasized in the discussion section.

RESPONSE:

Thank you for your thoughtful and constructive advice to strengthen the quality of our manuscript. As per your suggestion, the related description of these results in the discussion section has been added to the revised manuscript at lines 379-386, as follows: *“We found that TMEM41B confers cell susceptibility to virus entry (Fig 4A–C) and cell permissively for CoV replication (Fig 4G–H). Although virion internalization level on TMEM41B KO cells were reduced, numerous virions successfully internalized into the cytoplasm (Fig 4C). However, we found that the internalized virions were unable to further replicate in TMEM41B KO cells (Fig 4G–H). The results strongly indicated that the replication stage of the virus depends the most on TMEM41B and may be the most biologically relevant stage. Our result was consistent with that of another report that showed that SARS-CoV-2 replication in cells lacking TMEM41B was reduced by more than 80% when the entry step was bypassed [28].”*

2. Lines 318-327 are discussion points and belong there, not in the results section.

RESPONSE:

Thank you for this valuable suggestion. As per your suggestion, the article has been restructured, especially discussion sections.

3. The text will need editing for syntax corrections.

RESPONSE:

Thanks for your suggestion. We feel sorry for our poor writing, the manuscript has been thoroughly revised by a native English speaker. And we hope the revised manuscript could be acceptable for you.

4. The figures can be clarified by matching color codes between panels, i.e., in Fig 7 and several other figs.

RESPONSE:

We thank the reviewer for pointing this out. We modified the Fig 3G, Fig 6G-H, Fig 7A-C and Fig. S11F-H (revised version) by matching color codes to make these figures were clarified and unified in manuscript.
